# Lesula: A New Species of *Cercopithecus* Monkey Endemic to the Democratic Republic of Congo and Implications for Conservation of Congo’s Central Basin

**DOI:** 10.1371/journal.pone.0044271

**Published:** 2012-09-12

**Authors:** John A. Hart, Kate M. Detwiler, Christopher C. Gilbert, Andrew S. Burrell, James L. Fuller, Maurice Emetshu, Terese B. Hart, Ashley Vosper, Eric J. Sargis, Anthony J. Tosi

**Affiliations:** 1 Lukuru Wildlife Research Foundation, Kinshasa, Gombe, Democratic Republic of Congo; 2 Division of Vertebrate Zoology, Yale Peabody Museum of Natural History, New Haven, Connecticut, United States of America; 3 Department of Anthropology, Florida Atlantic University, Boca Raton, Florida, United States of America; 4 Department of Anthropology, Hunter College of the City University of New York, New York, New York, United States of America; 5 New York Consortium in Evolutionary Primatology, New York, New York, United States of America; 6 Center for the Study of Human Origins, Department of Anthropology, New York University, New York, New York, United States of America; 7 Department of Ecology, Evolution and Environmental Biology, Columbia University, New York, New York, United States of America; 8 Wildlife Conservation Society Zanaga Project, Wildlife Conservation Society Congo, Brazzaville, Republic of Congo; 9 Department of Anthropology, Yale University, New Haven, Connecticut, United States of America; Zoological Society of London, United Kingdom

## Abstract

In June 2007, a previously undescribed monkey known locally as “lesula” was found in the forests of the middle Lomami Basin in central Democratic Republic of Congo (DRC). We describe this new species as *Cercopithecus lomamiensis* sp. nov., and provide data on its distribution, morphology, genetics, ecology and behavior. *C. lomamiensis* is restricted to the lowland rain forests of central DRC between the middle Lomami and the upper Tshuapa Rivers. Morphological and molecular data confirm that *C. lomamiensis* is distinct from its nearest congener, *C. hamlyni*, from which it is separated geographically by both the Congo (Lualaba) and the Lomami Rivers. *C. lomamiensis,* like *C. hamlyni,* is semi-terrestrial with a diet containing terrestrial herbaceous vegetation. The discovery of *C. lomamiensis* highlights the biogeographic significance and importance for conservation of central Congo’s interfluvial TL2 region, defined from the upper Tshuapa River through the Lomami Basin to the Congo (Lualaba) River. The TL2 region has been found to contain a high diversity of anthropoid primates including three forms, in addition to *C. lomamiensis*, that are endemic to the area. We recommend the common name, lesula, for this new species, as it is the vernacular name used over most of its known range.

## Introduction

**Figure 1:**
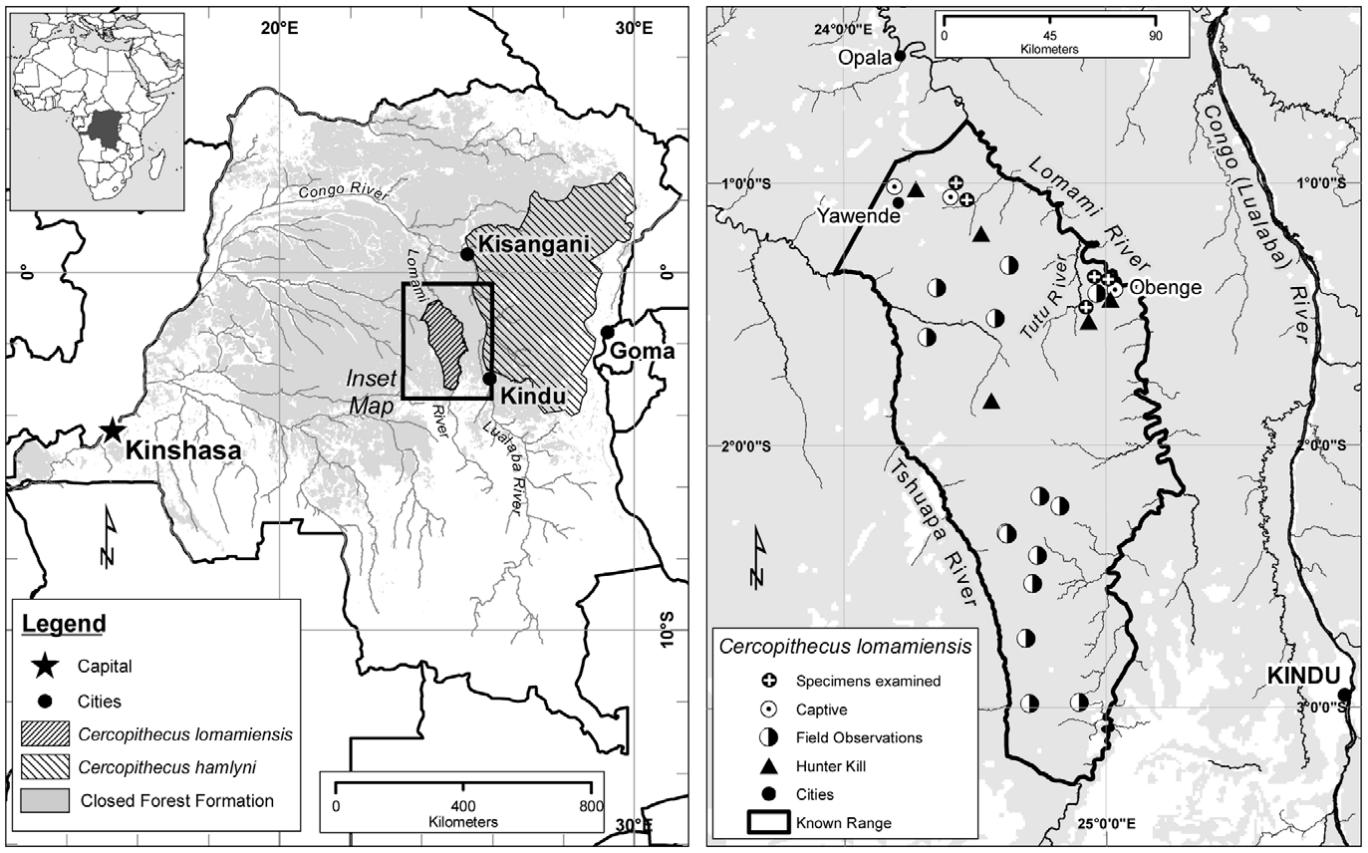
Map of study area. Distribution of *Cercopithecus lomamiensis* (sp. nov.) and its sister species, *C. hamlyni* (left), and locations of specimens and observations of *C. lomamiensis* (right). Outside of DRC, *C. hamlyni* occurs only at Nyungwe Forest National Park, Rwanda. The TL2 region extends from the upper Tshuapa River, across the Lomami River to the Lualaba River. See Table SI for details of specimens cited.

Discoveries of new African primate species are rare but significant events that clarify taxonomic and evolutionary relationships and highlight important regions of biodiversity for conservation. Here we report the scientific discovery of a new primate species, *Cercopithecus lomamiensis*, sp. nov., found during field surveys in a remote area of the middle Lomami Basin in central Democratic Republic of Congo (DRC) (Fig. 1). *C. lomamiensis* represents only the second new species of African monkey to be discovered in the past 28 years. The new species is a member of the tribe Cercopithecini, commonly referred to as guenons, which represents the most speciose clade of extant African primates. Guenons are endemic to sub-Saharan Africa and occupy a range of habitats from wooded savannas to closed forest [1]. The highest diversity of guenons occurs in closed forests in Central and West Africa where species utilize different canopy levels, including the forest floor [2], and exhibit considerable dietary flexibility, exploiting a diversity of leaf-, insect- and fruit-eating rainforest niches [3]. Geographical and behavioral barriers have been potentially important in guenon speciation [4], [5], and the distribution and relationship of species in related clades provides insight into the biogeographic history of Central African faunas and the evolution of key behavioral and ecological traits.

In this paper, we describe and name the new guenon species and discuss its relationship with its nearest congener and sister species, *Cercopithecus hamlyni*. This discovery adds a new species to the previously monotypic and poorly known *hamlyni* species group, and expands our understanding of this unique, semi-terrestrial lineage within the guenon radiation. It furthermore highlights the biogeographic significance and importance for conservation of the eastern interfluvial region of the Congo River’s central basin, known as the TL2 landscape, from the upper Tshuapa through the Lomami River Basin to the Congo (Lualaba) River (Fig. 1). This previously little surveyed forest region is shown to have high taxonomic richness and endemism of anthropoid primates and represents an important area for conservation of Central African forest faunas.

## Materials and Methods

### 1. Ethics Statement

The Congolese Wildlife Authority (Institut Congolais pour la Conservation de la Nature, ICCN) issued permits to the TL2 (Tshuapa, Lomami, and Lualaba) Project for all sites where biological samples and field observations were made. The ICCN is the governmental authority that has jurisdiction over the wildlife of this territory. Institutional Animal Care and Use Committees (IACUC) approval was not required for the noninvasive behavioral observations and biological samples of wild monkeys used in this study. IACUC protocols were followed for the collection of one skin snip specimen from a captive monkey. For the specimens collected from hunted animals, we obtained approval from the hunters to use these samples and no animal was hunted for the purpose of research. We acquired specimens only opportunistically in villages outside of the forest and we did not request samples from all lesula available to avoid targeting this species. When we encountered captive monkeys in villages, we photographed them with permission from the owner. We advised owners on the monkeys’ care and discouraged owners to acquire wild animals as captives. All the necessary exportation and importation permits were acquired by CITES, Centers for Disease Control and Prevention and the U.S. Fish and Wildlife Services.

### 2. Specimens Examined

Seven specimens of *C. lomamiensis* and eight specimens of *C. hamlyni* were used for analyses and descriptions of *C. lomamiensis* and *C. hamlyni* (Tables S1, S2). Specimens collected in the field included freshly killed animals acquired from local hunters, animals killed by predators (including kills by leopards, *Panthera pardus*, or crowned eagles, *Stephanoaetus coronatus*) and one skin snip from a monkey captured locally and kept as a captive in a village near the species’ range. We used GPS to record locations where specimens were recovered in the field; when exact location of specimen origin was not possible (e.g., location based on hunter reporting), locations were estimated to the nearest settlement or geographic feature. We took information on the provenance, history and care of all captive animals seen. We took photographs of all specimens and captives, and recorded standard field measurements (total length, tail length, length of hind foot, length of ear pinnae, and body mass) wherever possible. Duplicates of tissue samples were stored in RNA*later* (Applied Biosystems, Foster City, CA) and 95% ethanol and stored in the field in cool, dark locations until transferred to the laboratory.

All *C. lomamiensis* skin and skeletal specimens are housed in the Yale Peabody Museum (YPM), New Haven, CT. Adult *C. hamlyni* specimens were examined at the YPM and the American Museum of Natural History (AMNH), New York, NY. See Table S1 for details on the specimens examined.

### 3. Morphology

Craniodental linear measurements were taken using digital calipers and recorded to the nearest tenth of a millimeter (Tables S3, S4). In addition to standard caliper measurements, we used 3-D geometric morphometric techniques to compare cranial shape of *C. lomamiensis* and *C. hamlyni*. The 23 landmarks chosen to capture the overall shape of the cranium are listed in Table S5 and include Type 1, Type 2, and Type 3 landmarks [6]; these landmarks included those used by Fleagle *et al.* [7] in their larger study of primate cranial diversity, and additional cranial landmarks deemed to be repeatable and informative.

We digitized the landmarks on each cranium using a Microscribe G2X digitizer (Immersion Corp). The individual crania were immobilized in a bed of Play-Doh, and the landmarks were digitized in two sets–one superior view and one inferior view. All bilateral landmarks were digitized on the right side of the cranium. As reference points, five landmarks were digitized in both views. Subsequently, the two sets of landmarks were combined using the program DVLR v. 0.4.9 [8] to obtain a single set of 23 three-dimensional coordinates for each cranium. We imported the landmark data into the software package Morphologika [9], and performed a Procrustes superimposition analysis on the entire sample of *C. lomamiensis* (n = 3) and *C. hamlyni* (n = 7) crania. We then used a principal components analysis (PCA) of the Procrustes superimposition to identify the major axes of cranial shape among all of the crania. Pelage and skin coloration are described using standard color references [10].

### 4. Genetics

#### 4.1. X- and Y-chromosomal loci

We assembled a ∼4.6 kb contig of X-chromosomal DNA from three overlapping amplicons for the newly surveyed *C. hamlyni* and *C. lomamiensis* individuals, using primers and protocols described by Tosi *et al*. [11]. The surveyed region is homologous to a portion of human Xq13.3 and consists solely of intergenic DNA [12]. This is a region of low recombination in both humans [13] and cercopithecins [11] and therefore unlikely to contain substantial reshuffling of ancestral DNA sequences, making it an excellent locus for phylogenetic study.

We amplified and sequenced a ∼2.2 kb segment of the Testis-Specific Protein, Y-chromosome (TSPY) using primers and protocols described by Tosi *et al*. [14]. TSPY is a multigene family located in the non-recombining portion of the Y-chromosome and is believed to have a function in spermatogonial proliferation [15]. Data already collected on this gene family suggest that it is maintained by a mechanism of concerted evolution in cercopithecine monkeys [11], [14].

#### 4.2. Sequencing and contig assembly

Amplified products were cleaned with exonuclease I and shrimp alkaline phosphatase [16] and cycle-sequenced using BigDye chemistry (Applied Biosystems, Foster City, CA). Cycle-sequence products were cleaned via ethanol precipitation and analyzed using an ABI 3730 automated DNA sequencer. Complementary strands were sequenced as a proofreading check of the data. The sequence reads from each amplicon were processed and assembled into a single contig using the program Sequencher v4.8 (Gene Codes Corp.).

#### 4.3. Data analysis

We used a phylogenetic analysis in a comparative context to address the question of species vs subspecies status for the *C. lomamiensis* individuals. If the divergence date calculated between the *C. lomamiensis* lineage and its sister lineage is equal to, or greater than, estimates calculated between recognized species within other cercopithecin species groups, it provides a strong argument for equivalent species-level status of the *C. lomamiensis* lineage. The TSPY and X-datasets were not combined for these analyses. X- and Y-loci differ in significant parameters, including effective population size and mode of inheritance, and are therefore known to follow unique evolutionary trajectories [17]. Moreover, earlier studies have shown that while the X-locus surveyed here is evolving in a clock-like fashion in cercopithecins [11], the TSPY gene is not [18].

The novel TSPY and Xq13.3 homolog sequences of *C. lomamiensis* and *C. hamlyni* were each added to datasets consisting of sequences of other cercopithecids taken from GenBank (Table S2). Both datasets were aligned with ClustalW2 [19] and checked by eye. Novel sequences developed in this study were archived in GenBank (Table S2). Alignments for each gene are provided in Matrix S1 and S2 as fully executable Nexus files. Appropriate models of molecular evolution were inferred using hierarchical likelihood ratio tests implemented in MODELTEST 3.6 [20]. The HKY + G model with an alpha value of 0.7748 was inferred for TSPY; TrN + G with an alpha value of 0.9416 was estimated for Xq13.3 homolog.

Maximum likelihood analyses using the appropriate models of molecular evolution were conducted first using PAUP* 4.0b10 [21] and then GARLI 0.951 [22], with 50 and 500 bootstrap replicates, respectively. *Trachypithecus cristatus* was used as the outgroup for the TSPY analysis, and *T. obscurus* for Xq13.3 homolog. GARLI allows much more rapid estimation of likelihood values and was therefore used to generate higher numbers of bootstrap replicates. Recommended default settings were used for the genetic algorithm implemented in GARLI.

Bayesian analyses of both datasets were conducted using MRBAYES 3.11 [23], [24] with the molecular evolutionary models inferred by MODELTEST. Each dataset was run twice with four chains for 1,000,000 generations and sampled every 100 generations. Sump and sumt burnins were 2500. Adequate mixing of chains and convergence of runs were verified using Tracer 1.5 [25].

#### 4.4. Molecular divergence date estimates

We used the BEAST 1.5.3 software package [26] to obtain estimates of dates of molecular divergences. This Bayesian program allows the inference of divergences despite significant rate variation among lineages. The XML input files were generated using BEAUti 1.5.3. The substitution models used in the BEAST analysis were again those inferred by MODELTEST (HKY for TSPY, GTR for Xq13.3 homolog), with base frequencies and alpha values estimated from datasets. The uncorrelated lognormal relaxed clock model was employed, with the rate of molecular evolution estimated from the data [27]. The analysis was started using the tree inferred by MRBAYES and with the tree prior set to the birth-death model. Operators were auto-optimized, and the analysis was run on Cornell University’s CBSU cluster (http://cbsuapps.tc.cornell.edu/index.aspx) with four repetitions of 20,000,000 (TSPY) or 50,000,000 (Xq13.3 homolog) MCMC generations logged every 1,000 generations. Several nodes were given priors based on estimated divergence (see Text S1 for additional methods on divergence date calibration).

The results of the four separate BEAST runs of both *C. lomamiensis* and *C. hamlyni* datasets were checked for adequate mixing and convergence using Tracer 1.5. Following successful convergence, BEAST tree files were combined using LogCombiner with burnins of 10,000 (TSPY) and 25,000 (Xq13.3 homolog). The combined sample of trees was summarized using TreeAnnotator 1.5.3 [26] before being visualized with Fig Tree 1.3.1 [26].

### 5. Behavior and Ecology

#### 5.1. Vocalizations

We recorded vocalizations of *C. lomamiensis* on the Losekola Study Area (S 1.38461°, E 25.03749°) between 1 April–25 April 2009 and of *C. hamlyni* near Epulu (N 1.40200°, E 28.57709°) in the Central Ituri Forest between 20 February and 3 March 2009. We concentrated recording sessions between about 05∶45 and 06∶30, a time when both species exhibit a marked increase in vocal behavior. Observers arrived at recording sites before dawn and remained stationary (and presumably undetected by the animals) throughout recording sessions.

We made recordings with a Marantz PMD 660 digital recorder equipped with a Sennheiser condenser microphone module (K6/K6P). All recordings were digitized with Raven 1.3 (Cornell Laboratory of Ornithology, Ithaca, New York) using a 1024-point fast-Fourier transform, Hanning window function. We used the resulting spectrographic displays to extract direct measurements of 11 spectral and temporal parameters and five calculated derived measures (Table S6).

#### 5.2. Dawn vocalization population surveys

Trained teams familiar with the calls of all the primates in the TL2 region conducted vocalization surveys between 13 March and 4 December 2009. Vocalization surveys were conducted at a total of 117 survey posts distributed systematically in six, 30 km×30 km blocks that had been previously selected for intensive large mammal inventories (18–22 vocalization surveys per block). The survey area included locations within *C. lomamiensis’* known range and adjacent forest east of the Lomami River and west of the Tshuapa River (Fig. 1). Listening posts were always ≥200 m from survey bivouac camps. Observers arrived at listening posts before first light and remained quiet and concealed during the survey. Observers recorded the species, time, compass direction, and relative distance of the calls for all primates heard between 06∶00–06∶30 in one of three distance categories: *proximate*: callers or their movements seen from the listening post; *far*: callers not visible but calls had high clarity and amplitude; and *remote*: all other discernible calls. For each sampling session, we estimated the minimum number of individual callers of each species using the direction and distance of recorded calls.

#### 5.3. Behavioral observations of unhabituated animals

We collected data on *C. lomamiensis* behavior on a pre-established path grid that totaled 64 km in the Losekola Study Area. We collected data over four sampling periods lasting from 10 days to three weeks from March 2008 to April 2010. Surveys were conducted in an area of diverse mature upland forest types bisected by small streams with no recent history of human settlement and little previous incidence of hunting. The Losekola Study Area was protected from all hunting since January 2008.

During survey periods, data were collected daily, from 6∶30 to 14∶00 by two experienced observers moving along the area’s permanent transect grid. Observers recorded the time and GPS waypoint for all primate groups encountered (seen and heard, or heard but not seen). For groups that were seen, we estimated the perpendicular distance from the estimated center of each to the transect line, counted the number of animals seen by species, and estimated the number of additional animals that could not be counted accurately. For groups that were heard but not seen, we recorded the occurrence of species based on vocalizations.

For each encounter with *C. lomamiensis,* observers noted the canopy position (vegetation stratum) and behavior of each visible animal when it was first detected. Stratum height position was recorded as: ground  = 0 m; understory  =  >0 m and <3 m; mid-story  = 3–20 m; canopy  =  >20 m. Behavior was recorded as stationary, including grooming and resting; feeding, including both ingesting and searching for food; slow movement, defined as movement where foraging was not detected; and rapid movement or flight. Animals still visible after 3 minutes were scanned a second time for position and behavior.

### 6. Nomenclatural Acts

The electronic version of this document does not represent a published work according to the International Code of Zoological Nomenclature (ICZN), and hence the nomenclatural acts contained in the electronic version are not available under that Code from the electronic edition. Therefore, a separate edition of this document was produced by a method that assures numerous identical and durable copies, and those copies were simultaneously obtainable (from the publication date noted on the first page of this article) for the purpose of providing a public and permanent scientific record, in accordance with Article 8.1 of the Code. The separate print-only edition is available on request from PLOS by sending a request to PLOS ONE, 1160 Battery Street, Suite 100, San Francisco, CA 94111, USA along with a check for $10 (to cover printing and postage) payable to “Public Library of Science”.

In addition, this published work and the nomenclatural acts it contains have been registered in ZooBank, the proposed online registration system for the ICZN. The ZooBank LSIDs (Life Science Identifiers) can be resolved and the associated information viewed through any standard web browser by appending the LSID to the prefix “http://zoobank.org/”. The LSID for this publication is: urn:lsid:zoobank.org:pub:7D4F3CF8-1C5C-4DC2-9F49-6DF2A1CDE217. This publication has been deposited in the following digital archives: PubMed Central, LOCKSS, Florida Atlantic University Institutional Repository.

## Results

### 1. Discovery

The scientific discovery of *Cercopithecus lomamiensis* was made in June 2007 when field teams saw a captive juvenile female of an unknown species at the residence of the primary school director in the town of Opala (S 0.50721°, E 24.22713°). The school director identified the animal as a “lesula” a vernacular name we had not recorded before, and said that it is well known by local hunters. He reported that he acquired the infant about two months earlier from a family member who had killed its mother in the forest near Yawende, south of Opala and west of the Lomami River (S 0.99772°, E 24.29810°). We took photographs of the animal and made arrangements for its care. We observed and photographed this animal regularly over the next 18 months.

Subsequent searches in Opala and in the Yawende area turned up other male and female captive juvenile lesula; all were photographed and some monitored for several months afterwards. Our first observation of the species in the wild was in the Obenge area (S 1.38461°, E 25.03749°) in December 2007 where the species is well known by local hunters.

### 2. Diagnosis and Description

***Cercopithecus lomamiensis*** J.Hart, Detwiler, Gilbert, Burrell, Fuller, Emetshu, T.Hart, Vosper, Sargis, and Tosi, **sp. nov.** urn:lsid:zoobank.org:act:8BA96F42-16A5-46B6-A194-B3DB0B2711B7.

#### Holotype

**Figure 2:**
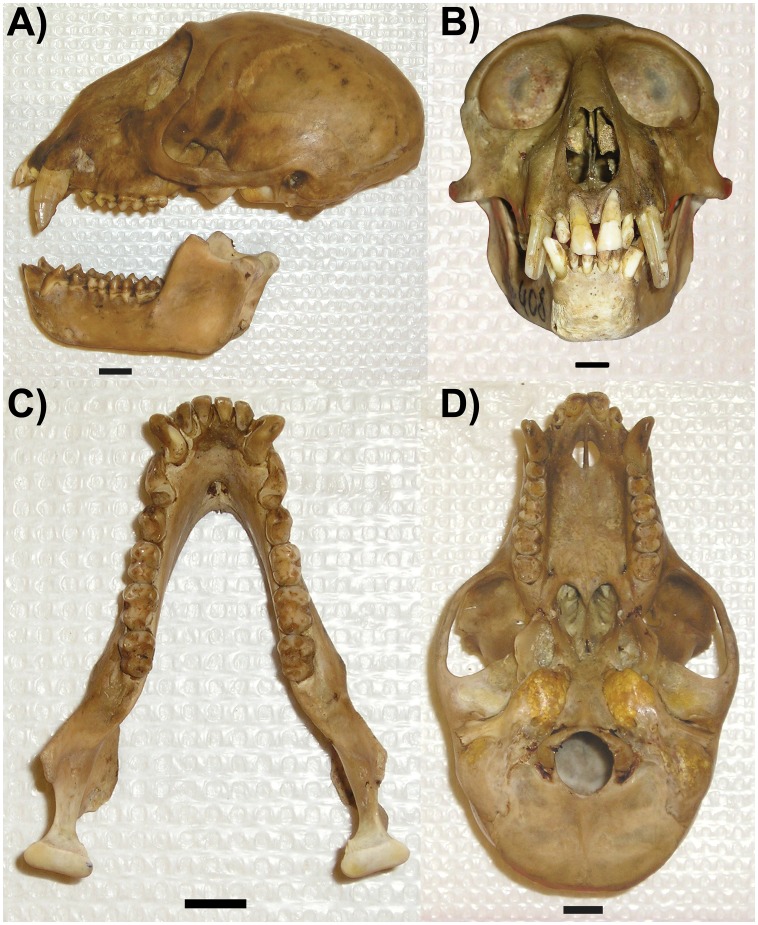
Skull of *Cercopithecus lomamiensis* (YPM 14080, holotype). Photographs show lateral (A) and anterior (B) views of the cranium and mandible, and occlusal views of the mandible (C) and cranium (D). Scale bar in each frame  = 1 cm.

YPM 14080, adult male, skin and skull (Fig. 2), collected near Lohumonoko, S 1.02237°, E 24.42368°, 470 m above sea level (asl). Housed at the Yale Peabody Museum.

#### Paratypes

YPM 14189, subadult female, skin and skull, collected in the forest near Yawende, S 1.06571°, E 24.44838°, 450 m asl. YPM 14190, subadult female, skin and skull, collected near the Lomami River, S 1.42801°, E 25.01601°, 415 m asl. YPM 14191, adult male, skin and skull, collected near Obenge Village, S 1.38145°, E 25.03843°, 420 m asl. YPM 14192, subadult female, skin and skull, collected west of Obenge, S 1.40129°, E 24.97498°, 460 m asl. See Table S1 for locality and field measures of specimens examined and Table S7 for brief descriptions of crania. Housed at the Yale Peabody Museum.

#### Type locality

West Bank, Lomami River (S 1.02237° to S 1.4280°, E 24.42368° to E 25.03843°), Democratic Republic of Congo (Fig. 1).

#### Diagnosis

**Figure 3:**
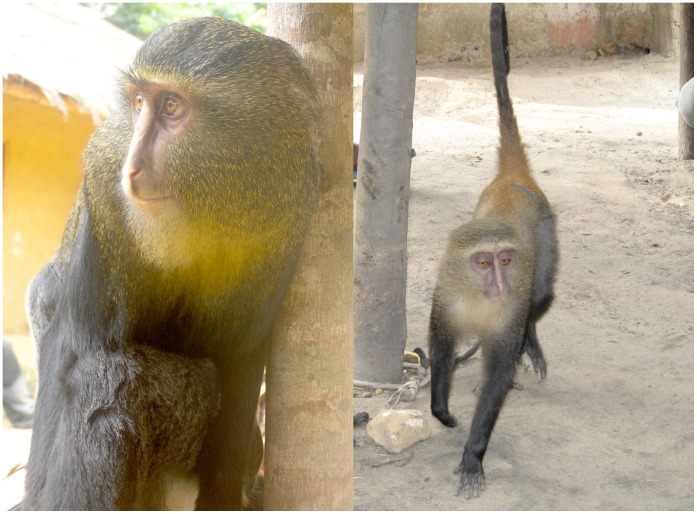
Captive *Cercopithecus lomamiensis*. Left: Adult male, Yawende, DRC. Photograph by M. Emetshu. Right: Subadult female, Opala, DRC. Photograph by J. A. Hart.

**Figure 4:**
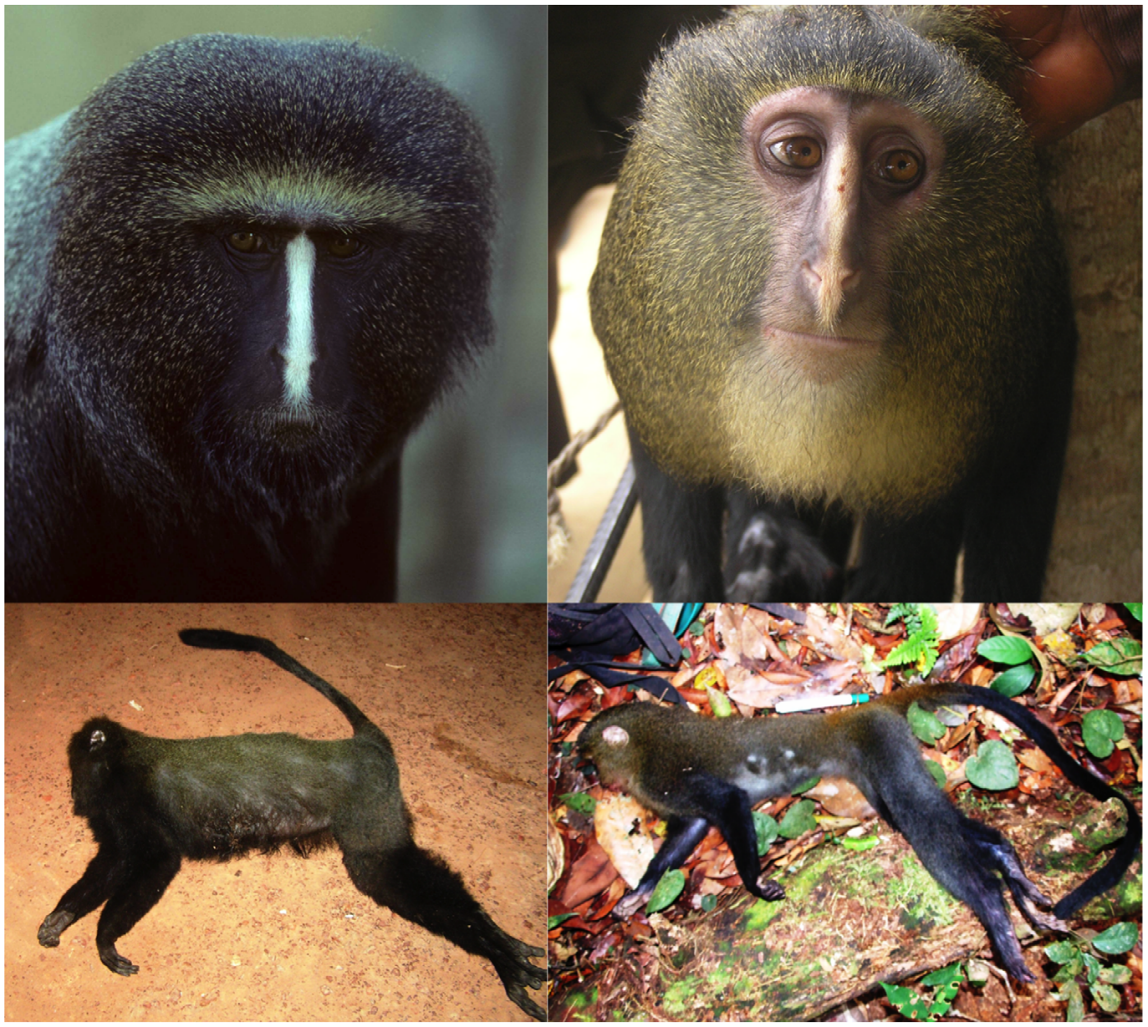
Adult pelage coloration. Portraits: Captive adult male *Cercopithecus hamlyni* (upper left), photo by Noel Rowe, with permission; and captive adult male *Cercopithecus lomamiensis* (upper right), Yawende, DRC, photo by Maurice Emetshu. Lateral view: Hunter-killed adult male *Cercopithecus hamlyni* (bottom left), photo by Gilbert Paluku; and eagle-killed subadult female *Cercopithecus lomamiensis* (bottom right), photo by Gilbert Paluku.

A mane of long grizzled blond hairs frames a protruding pale, naked face and muzzle, with a variably distinct cream colored (color 54 [10]) vertical nose stripe (Figs. 3, 4). An amber (color 36 [10]) median dorsal patch, brightest at the base of tail, contrasts with pale upper ventrum and black lower limbs and tail (Figs. 3, 4). Black hair on the dorsum is banded with 3–4 buff (color 124 [10]) or amber bands.

**Figure 5:**
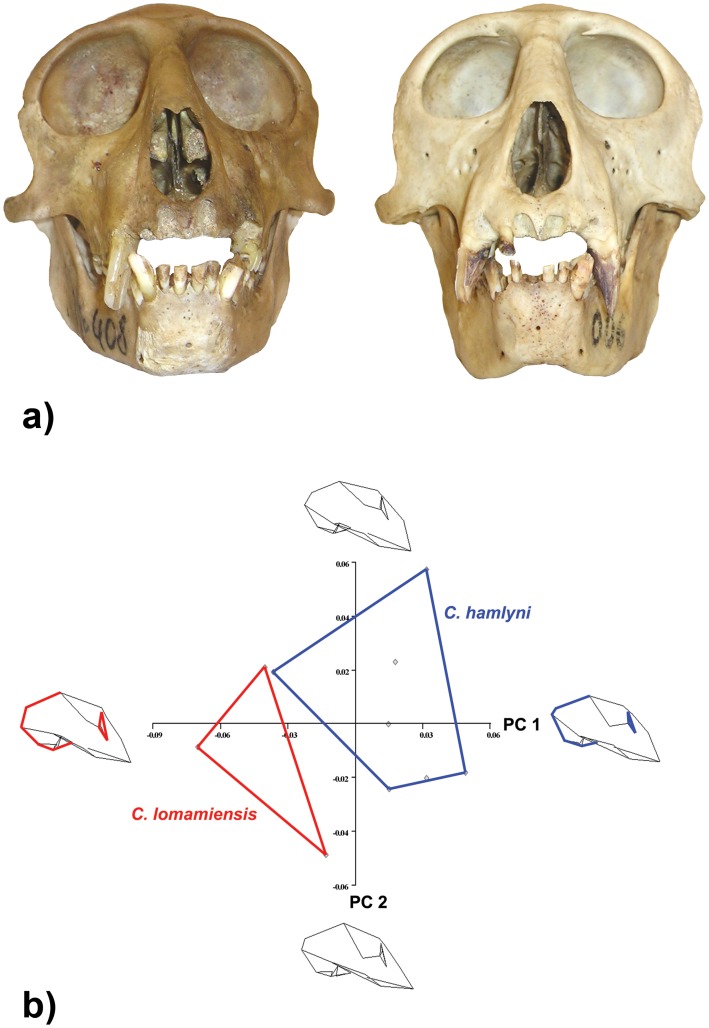
Cranial morphology. Comparative crania of *C. lomamiensis* (left) and *C. hamlyni* (right) (A), and principal components analysis of Procrustes aligned coordinates in a 3-D geometric morphometric analysis of 23 landmarks on cercopithecin crania (B). The wireframe crania at each end of PC1 and PC2 represent the shape changes observed moving across a given axis. Distinctive changes in cranial shape separating the taxa on PC1 are associated with orbit size and occipital length/flexion. The minimum convex polygon representing *C. lomamiensis* (n = 3) does not overlap with the minimum convex polygon representing its sister species, *C. hamlyni* (n = 7). See Table S4 for details of statistical results.

Cranium with a distinctive elongated nasal profile and large orbits (Figs. 2, 5). The face (nasion-prosthion) is relatively klinorhynch, angled downward relative to nasion-basion at approximately 85–92 degrees. The cranium of *C. lomamiensis* differs from *C. hamlyni*, having significantly larger orbits, an elongated occipital region (as characterized by the lambda-inion chord), a narrower interorbital region, a greater degree of occipital flexion, and a narrower calvarium (Fig. 5, Table S4). Dentally, *C. lomamiensis* differs from *C. hamlyni* in its significantly larger incisors, upper and lower second molars, and upper M3s (Table S4).

#### Description

A medium sized, long-limbed monkey with a slender body. Naked facial skin, eyelids, and ear pinnae vary from pale pinkish gray to tannish brown. Buff diadem and vertical cream nose stripe are variably present. Chin, throat, and upper ventrum are yellowish buff, contrasting with black lower ventrum and abdomen. Skin and pelage on shoulder and forelimb are black. Hair over the entire dorsum is banded buff or amber with black. Typically, the base of the hair grades from white to gray to black and then exhibits 3–4 bands of buff/amber as one moves up the hair distally/dorsally. Anterior 2/3 of the dorsum, including head and mane, is buff, grading into amber over posterior third of dorsum and onto base of tail. Distinctively, there is a prominent amber median stripe on the distal half to one-third of the dorsum. The amber banding is restricted to the medial portion of the posterior third to half of the dorsum (see Fig. 3). Laterally there is buff banding similar to the buff banding on the proximal half to two-thirds of the dorsum. The proximal half of the thigh is characterized by silvery-gray hairs, slightly lighter than color 86 [10], while the rest of the leg (underside and distal half) is covered with black hairs. Proximal end of tail is amber, darkening distally and becoming completely black at its tip.

**Figure 6:**
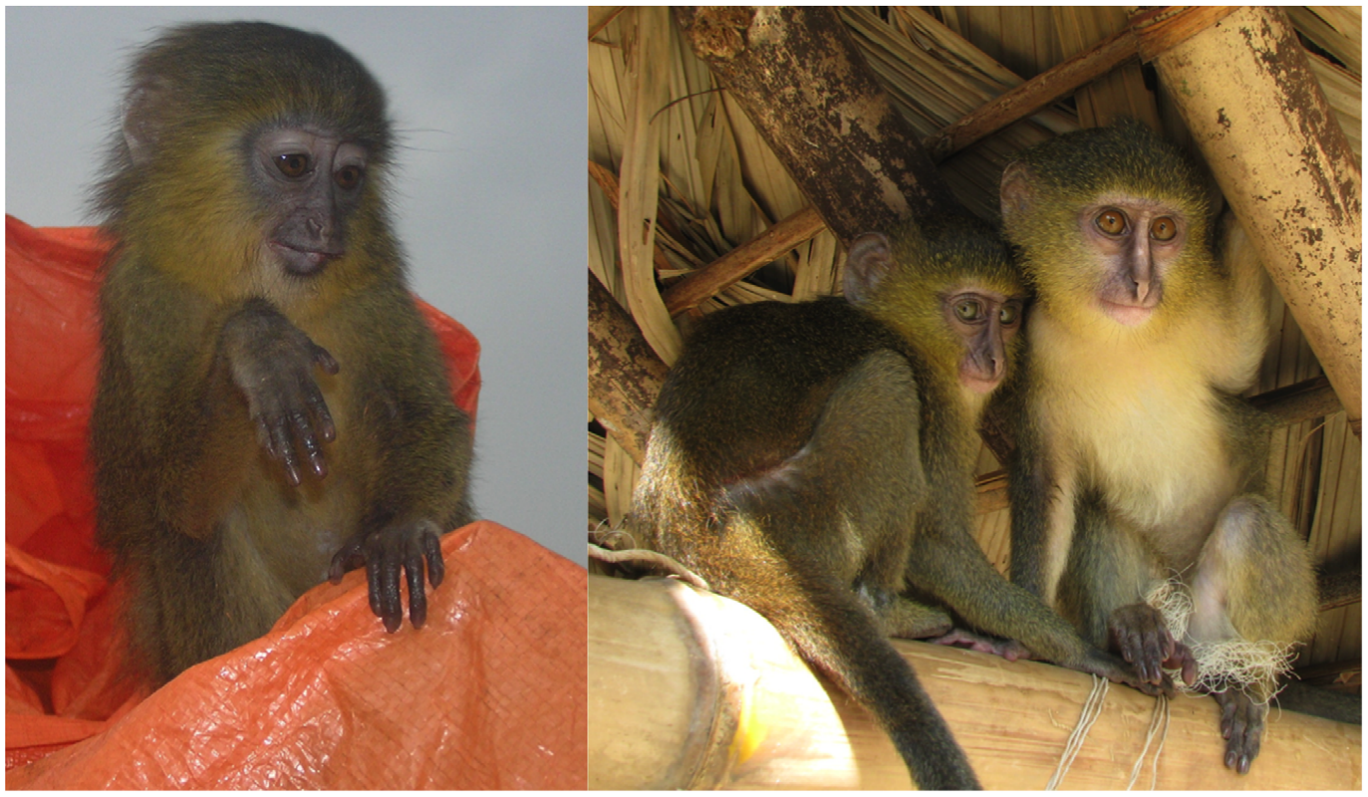
Juvenile coloration. *Cercopithecus hamlyni*, captured east of Kisangani, DRC (left), and *Cercopithecus lomamiensis*, captured near Obenge, DRC (right). White nose stripe is variably present in juvenile *C. hamlyni* from the Kisangani region. Photos by John Hart.

Juveniles have a pale blond pelage overall, lightest on the throat and upper ventrum (Fig. 6). Vertical nose stripe is absent or reduced. The amber dorsal patch appears on the lower dorsum and base of the tail within the first three months. Dorsal and limb pelage darken progressively. One female followed from an estimated age of 3 months acquired adult pelage and full adult body size by about 15 months of age.

**Figure 7:**
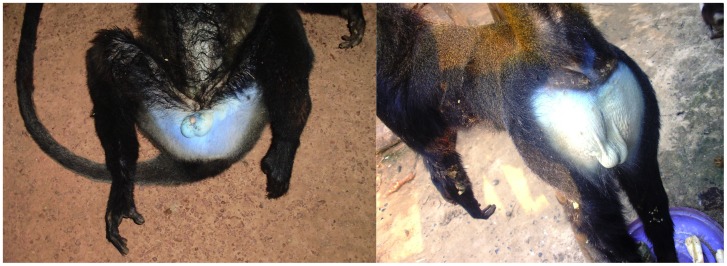
Adult male perinea and scrota. Hunter-killed *C. hamlyni* (left) and hunter-killed *C. lomamiensis* (right). Photos by Gilbert Paluku.

**Figure 8:**
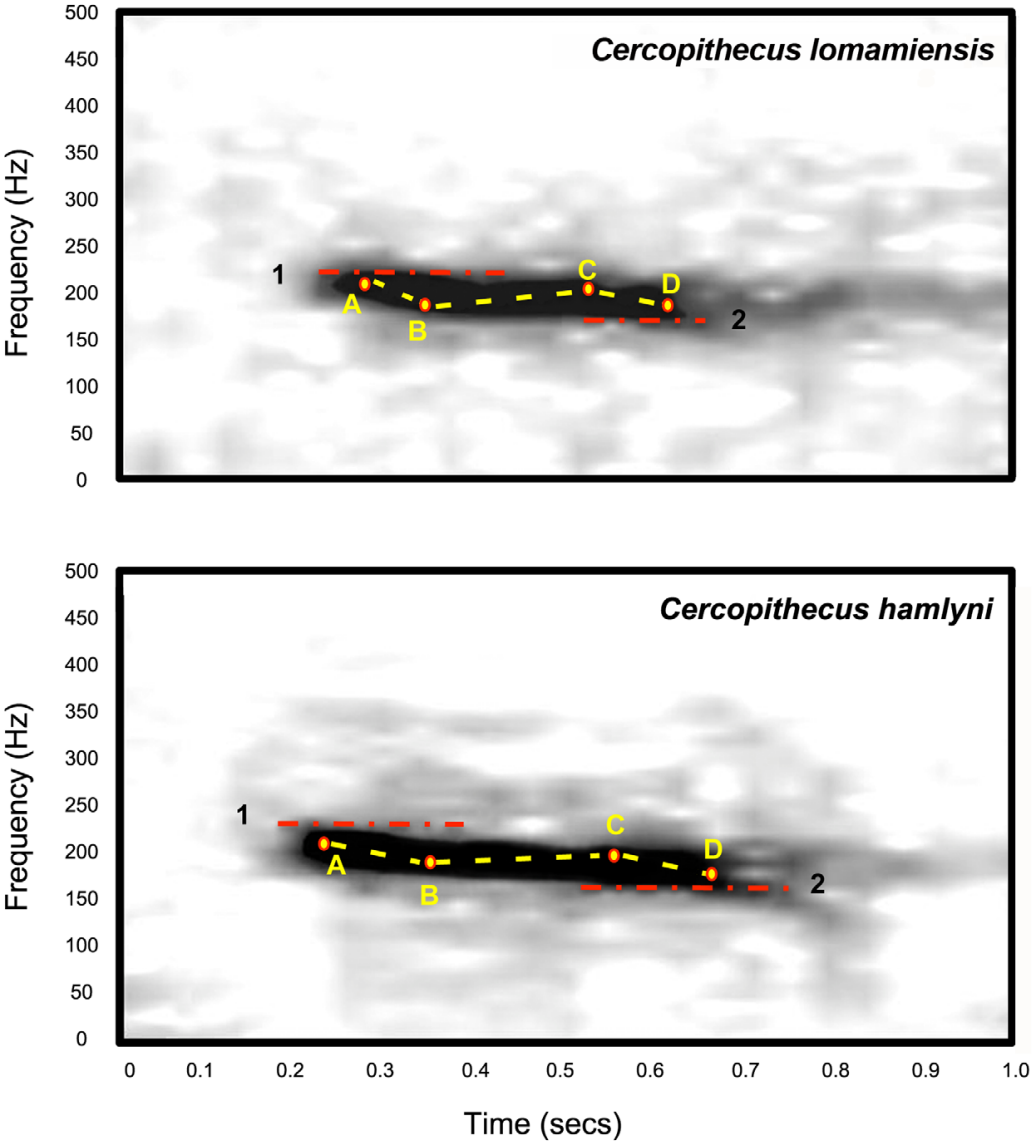
Spectrograms of booms of *C. lomamiensis* and *C. hamlyni*. Frequency (Hz) is on the Y-axis; time (sec) is on the X-axis. Mean values for acoustic measures for each species are indicated: 1. High frequency, 2. Low frequency, A. Start frequency, B. Q1 frequency, C. Q3 frequency, D. End frequency, dotted line: slopes.

Sexual dimorphism is present for body and canine size, with the adult male larger. An adult male exhibits a bright blue scrotum and perineum (Fig. 7), which fade quickly upon death becoming creamy white in dried skins. The male *C. lomamiensis* emits a characteristic low frequency, descending, loud call or boom similar to, but distinguishable from that of *C. hamlyni* (Fig. 8, Table S8, Audio S1 and S2).

#### Measurements

Adult males (n = 2): head and body length 47–65 cm, weight 4.0–7.1 kg. Subadult females (n = 2): head and body length 40–42 cm, weight 3.5–4.0 kg. See Table S1 for additional measurements.

#### Etymology

The specific name acknowledges the Lomami River near which the type specimen was found and which traces the eastern boundary of the species’ range.

#### Distribution

*C. lomamiensis* is restricted to about 17,000 km^2^ of forests in DRC’s eastern central basin (Fig. 1). Its known range is bordered on the east by the Lomami River (25°E) in Maniema and Orientale Provinces and on the west by the upper Tshuapa River (24°E) in Sankuru District of Kasai Oriental Province. *C. lomamiensis* ranges south to the limits of the forest at approximately 3° 30′ S. Its northern limits are not well established, but we have no report of the species occurring north of 1° S. Elevations in the distribution of *C. lomamiensis* vary from 400 to 715 m.

#### Habitat

Occurs in mature terra firma evergreen forests.

#### Vernacular names

Lesula (Kingengele, Kilanga, Kimbole), Kifula (Kinyamituku), Tou (Kitetela).

#### Extended description and comparisons with *C. hamlyni*

Similarities in cranial, skin and pelage traits between *C. lomamiensis* and *C. hamlyni.*

The cranium of *C. lomamiensis* is similar to that of *C. hamlyni* in possessing a distinctive nasal profile (adult males, in particular) that distinguishes crania in this clade from other *Cercopithecus* species. On the basicranium, *C. lomamiensis* and *C. hamlyni* both seem to exhibit distinctive and well-excavated fossae anterior to the foramen magnum (for longus capitis).

The variably prominent white to cream colored vertical nose strip shared by *C. hamlyni* and *C. lomamiensis* is found in no other *Cercopithecus* species. The pelage of both species includes hairs that are gray at the base with light and dark band pairs moving distally. The limbs of both species, including the back of the thighs and perianal region, are black with admixture of gray/silvery hairs. Males and females of both *C. lomamiensis* and *C. hamlyni* have a prominent facial mane of elongated hairs on the cheeks and crown, a feature unique to these two species.

The blue perineum, buttocks and scrotum displayed by adult males are comparable in size and coloration in *C. lomamiensis* and *C. hamlyni* (Fig. 7), and are more extensive than genital patches in any other *Cercopithecus*. It is comparable in extent to the buttocks and genital patch displayed in adult male mandrills (*Mandrillus sphinx)*.

In both species, the juvenile is pale, uniformly colored and differs markedly from adults (Fig. 6). The nose stripe is variably present in juveniles of both species, and is often absent or indistinct.

### Differences in Cranial, Skin and Pelage Traits between *C. lomamiensis* and *C. hamlyni*

Overall, the olive-maned *C. hamlyni* is darker, more somber and less vividly marked than *C. lomamiensis* whose grizzled blond mane, prominent amber dorsal patch, and buff upper ventrum contrast with black legs and lower ventrum. The crown hairs of *C. lomamiensis* are generally not offset by a contrasting diadem as is often seen in *C. hamlyni*. The pale facial skin and ear pinnae of *C. lomamiensis* are distinguished from the dark facial skin and pinnae of *C. hamlyni.* The nose stripe is typically a yellowish/cream color and often indistinct in *C. lomamiensis* rather than the clear white of *C. hamlyni.* Typically, there are 3–4 bands on the dorsum hairs of *C. lomamiensis* and 4–5 bands on the hairs of *C. hamlyni* [28]. The gray/silver hairs on the thighs of *C. lomamiensis* do not extend as far distally as in *C. hamlyni*. The tail of *C. lomamiensis* is amber at its base, black over most of its length and lacks a terminal tail tuft. The tail of *C. hamlyni* is gray from the base to over 3/4 of its length and is only black at its tufted terminal tip (Fig. 4).

The juvenile pelage of *C. lomamiensis* is distinctively paler and blonder than that of *C. hamlyni*. Juvenile *C. lomamiensis* have pale facial skin in contrast to the darker facial skin of *C. hamlyni*, and they show the beginnings of the amber dorsal patch even when immature (Fig. 6).

The cranium of *C. lomamiensis* is most obviously distinguished from *C. hamlyni* by its significantly larger orbits, greater degree of occipital flexion, narrower interorbital breadth, and narrower calvarium (Fig. 5, Table S4). *C. lomamiensis* has significantly larger incisors, upper and lower M2s, and upper M3s than *C. hamlyni* (Table S4). In addition, a distinct bump or prominence is typically present at or around nasion in *C. hamlyni*, but is absent in the type specimen of *C. lomamiensis.*

### 3. Genetics

**Figure 9:**
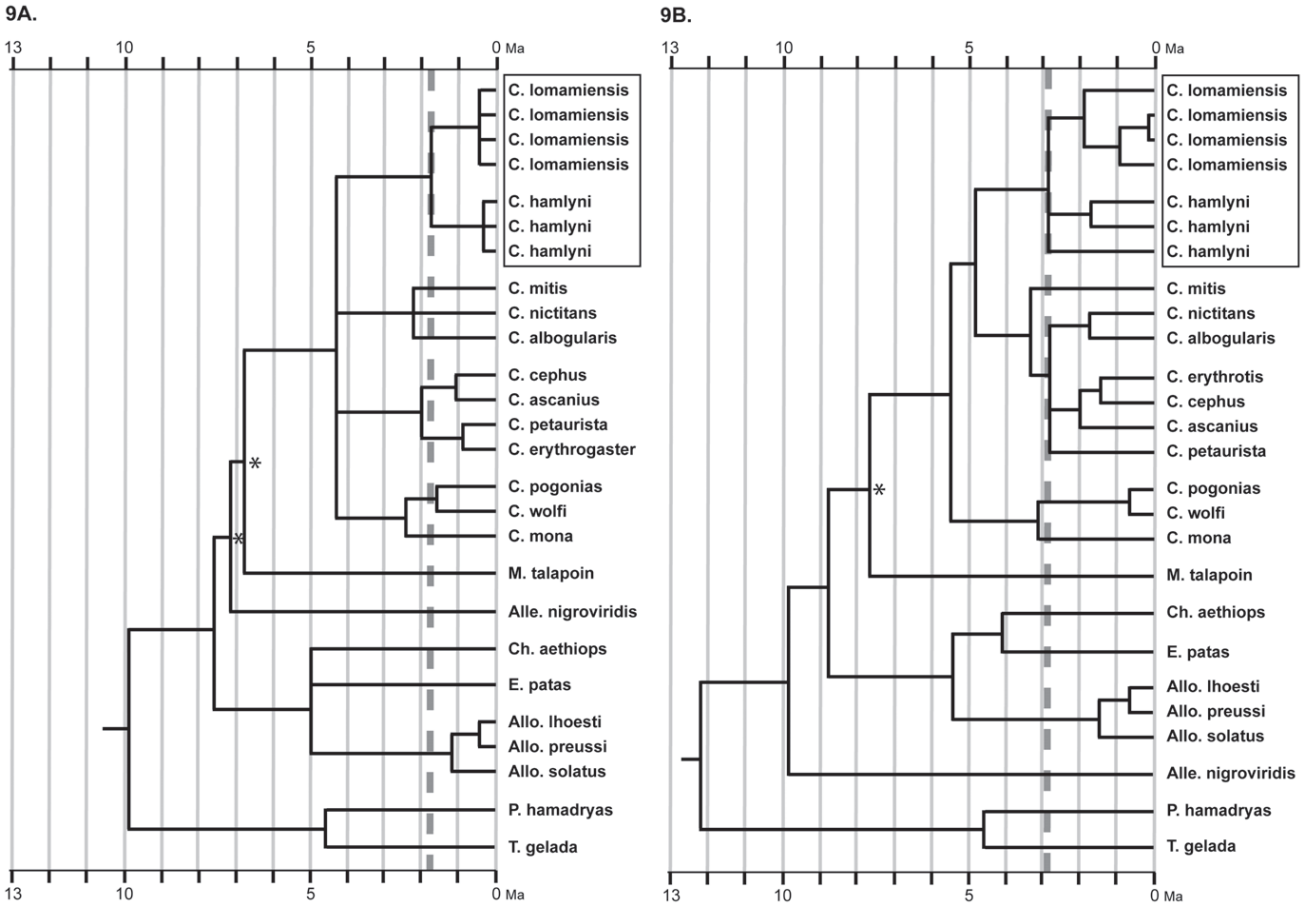
Estimated cercopithecin divergence dates. Dashed gray lines highlight the inferred divergence date between *C. lomamiensis* and *C. hamlyni* for each locus: TSPY (A), ∼ 1.7 Ma (3.2–0.5 Ma), and Xq13.3 homolog (B), ∼ 2.8 Ma (4.3–0.6 Ma). For both loci, mean divergence date estimates were inferred using a Bayesian approach implemented in BEAST 1.5.3. Confidence intervals of 95% for all nodes are given in Table S9. Tree topology follows that inferred by maximum likelihood and Bayesian analyses using GARLI and MRBAYES, respectively. Clades supported by ML bootstrap and Bayesian clade credibility values of <90/0.90 are marked with *.

Maximum likelihood and Bayesian phylogenetic analyses of TSPY sequence data clearly show that *C. hamlyni* and *C. lomamiensis* are reciprocally monophyletic and form a clade exclusive of the other cercopithecins (Fig. 9A, Figures S1, S2). Similar analyses of the Xq13.3 homolog show all *C. lomamiensis* individuals cluster together. The relationship of one *C. hamlyni* sample to this clade, however, could not be fully resolved (Fig. 9B, Figures S3, S4) possibly due to a lack of enough synapomorphic mutations to resolve the relationship, or the retention of an ancestral X-lineage in this *C. hamlyni* individual. Yet, this topology remains consistent with reciprocal monophyly between *C. hamlyni* and *C. lomamiensis*, as seen in the TSPY tree.

**Figure 10:**
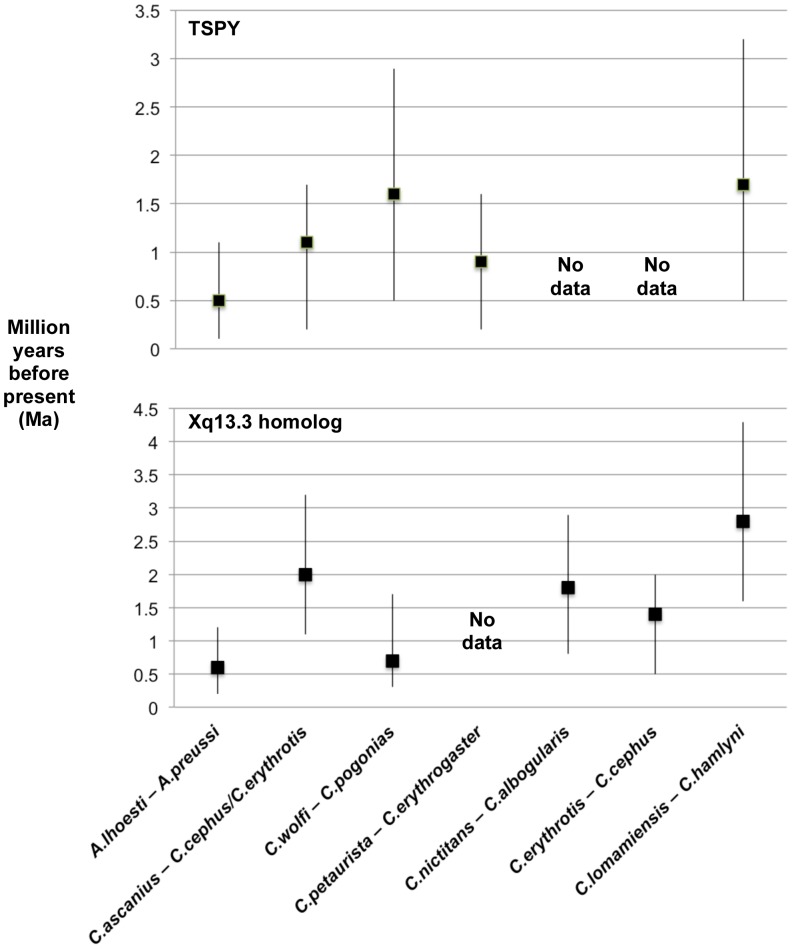
Estimated divergence dates (Ma) for selected guenons (*Cercopithecus*) based on analyses of TSPY and Xq13.3 homolog sequences. Values shown are mean and 95% Confidence Interval for estimates. See Table S9 for full comparisons.

Molecular divergence date estimates of the most recent common ancestor (MRCA) of the *C. lomamiensis–C. hamlyni* clade is inferred at ∼1.7 Ma (95% confidence interval 3.2–0.5 Ma) for TSPY and ∼2.8 Ma (95% confidence interval 4.3–1.6 Ma) for the Xq13.3 homolog (Figs. 9, 10, Table S9). The older MRCA estimates for the X-dataset likely reflect the presence of ancestral alleles and/or deeper coalescence at the X-locus. Molecular estimates of the divergence between *C. lomamiensis* and *C. hamlyni* are similar to, or predate divergences between, well-recognized species within other cercopithecin species groups (Fig. 10).

### 4. Vocalizations

The dawn boom chorus is the most conspicuous vocalization of both *C. lomamiensis* and *C. hamlyni* and the only call regularly heard in free-ranging animals. We consistently heard booms of *C. lomamiensis* around dawn and very little during the remainder of the day. We heard occasional booms by *C. lomamiensis* at night, most often shortly after dark. Though we did not identify the sex of callers, they were almost certainly male; several other *Cercopithecus* species (e.g., *C. campbelli*, *C. neglectus*, *C. mitis*, *C. wolfi*) make similar boom calls utilizing a laryngeal air sac that is substantially larger in males and in no species do females produce such a call [29]. We collected 49 useable recordings of ‘descending booms’ by *C. hamlyni* (n = 43) and *C. lomamiensis* (n = 6). No other call types were recorded. Exemplars of booms by *C. hamlyni* and *C. lomamiensis,* filtered for calls of birds and insects and other ambient sounds are provided in Audio S1 and S2.

The booms of *C. lomamiensis* and *C. hamlyni* are low frequency, tonal calls clearly distinguished from booms in other *Cercopithecus* species by their longer duration and descending frequency from beginning to end. In both species, booms were given in bouts of 2–3 calls and different animals often called in overlapping sequences, suggesting contagion or potentially antiphonal calling. The booms of both species consist of a single dominant frequency band with no discernible harmonic overtones or formants (Fig. 8). Booms have a mean starting frequency of 215 Hz in *C. lomamiensis* and 205 Hz in *C. hamlyni* and a mean end frequency of 199 Hz in *C. lomamiensis* and 188 Hz in *C. hamlyni*. Call duration averaged 0.35 sec in *C. lomamiensis* and 0.37 sec in *C. hamlyni*.

The calls differed significantly (Welch Two Sample t-test, n = 49, p<0.05) on two of 16 measured acoustic parameters: High frequency and 3^rd^ Quartile Frequency Differences. Three other parameters (End Frequency, Start Frequency, and 1^st^ Quartile Frequency) were nearly significant (range: p = 0.054–0.065) (Table S8).

### 5. Ecology

We found *C. lomamiensis* in mature upland evergreen humid forests, including mixed forests and forests dominated by *Gilbertiodendron dewevrei*. We recorded *C. lomamiensis* less often in regenerating forests around settlements. We never recorded *C. lomamiensis* in seasonally inundated forests or in the gallery forests in the savannas south of the main forest block.

*C. lomamiensis* is shy and was the least frequently seen of all primates recorded on large mammal surveys (19 observations of *C. lomamiensis* out of a total of 223 visual observations of primate groups). Vocalization surveys indicate that *C. lomamiensis* is more abundant than visual sightings on transects would suggest. We recorded *C. lomamiensis* boom calls at 34 of 70 dawn listening posts surveyed within the species’ known range between the Lomami and upper Tshuapa Rivers. We surveyed an additional 47 listening posts outside the known range, east of the Lomami River and west of the Tshuapa River and recorded no *C. lomamiensis* vocalizations.

Multiple individuals were recorded calling at 27 of 34 (79.4%) sites where *C. lomamiensis* vocalizations were heard. Of the 27 posts with multiple animals calling, two vocalizing individuals were recorded at 17 posts; three vocalizing individuals were recorded at 4 posts; 4 vocalizing individuals at 5 posts; and 5 vocalizing individuals were recorded at one post. When multiple animals vocalized, it was not possible to determine if the animals belonged to different groups or were members of a single dispersed group. Of 78 calling individuals, five callers were classed as proximate. Two proximate vocalizing animals were seen on the ground; three others were detected by moving vegetation, either on the ground or in the understory. All other vocalizing animals were classed as far or remote.

We found no evidence for seasonal variation in calling rates; however, we found marked differences in the occurrence of calling in different portions of the species known range. We recorded vocalizations at 11 of 40 (27.5%) listening points in the southern third of the range. In contrast, vocalizing animals were recorded at 16 of 19 listening points in the region of the Losekola Study Area and at 8 of 11 listening points in the northern portion of the species range. These latter two locations are in the Tutu River basin, a tributary of the Lomami River. These apparent differences in occurrence of *C. lomamiensis* may be due to habitat differences in the areas surveyed. The southern range has lower rainfall with a pronounced dry season and a higher frequency of savanna ecotone, where lesula were never recorded. Closed evergreen forests dominate the northern range of *C. lomamiensis*, including the Tutu River basin.

### 6. Behavior

We observed 48 individual *C. lomamiensis* during 19 encounters (1–5 individuals per encounter), with only a single animal seen in 8 of the encounters. In 11 encounters, *C. lomamiensis* were seen associated with other primate species (*C. mona wolfi*, *C. ascanius katangae*, *Procolobus badius tholloni*). Of the 48 animals seen, 17 (35%) were on the ground when first encountered.

Fourteen animals fled at or subsequent to encounter. Nine animals fled on the ground including three individuals that descended to the ground from the understory or middle level stratum to flee. Of the five animals fleeing in the strata above ground, only one climbed from the ground to flee. One animal first seen in the canopy fled laterally to a second canopy and then became stationary. In the other three observations, animals located above the ground fled in the same stratum in which they were first encountered.

Animals were recorded feeding or foraging on eight first encounters totaling 26 individuals. In five observations of feeding (20 animals total) *C. lomamiensis* were associated with other primate species; in three encounters (6 animals total) *C. lomamiensis* were in a monospecific group or only a single animal was seen. Eleven of the 26 individuals observed feeding were feeding on the ground. In two encounters of multi-species associations, we observed *C. lomamiensis* feeding on the ground on food parts dropped by primates foraging above them in the middle story and canopy. We observed *C. lomamiensis* feed on leaf petioles, ripe and unripe fruit parts, flower buds, and the meristems of two species of terrestrial herbaceous Marantaceae.

We made one exceptional observation of an apparent attack by a crowned eagle (*Stephanoaetus coronatus*) on a lesula. One of our survey leaders saw an eagle fly off at close range in mid story. We approached the site and saw a *C. lomamiensis* alive but wounded lying in a tree crotch. We watched the site for 37 minutes but the eagle did not return. At that point, the monkey, a subadult female, died and fell out of the tree and the animal was collected (specimen YPM 14192).

## Discussion

We applied a comprehensive approach to assess the evolutionary distinctiveness and appropriate taxonomic naming of the new primate discovery [30]. We conducted field studies of behavior, ecology and range determination in the DRC as well as morphological and genetic assessments. Our results unambiguously identify the new primate as a distinct species, *Cercopithecus lomamiensis*.

### 1. *C. lomamiensis* and its Sister Species

Morphological and genetic analyses support the species designation of *C. lomamiensis* and place it with *C. hamlyni* in a lineage divergent from related clades of *Cercopithecus*. Phenotypic and behavioral traits that are shared by *C. hamlyni* and *C. lomamiensis* and that are found in no other *Cercopithecus* species include a distinctive skull shape, prominent facial mane of elongated hairs on the cheeks and crown found in both sexes, variably white to cream colored vertical nose stripe, large bright blue patch complete with a blue scrotum on the perineum and buttocks in adult males, and dawn boom chorus. A pale, uniformly colored juvenile pelage differing markedly from adults is found in both *C. hamlyni* and *C. lomamiensis* and is shared with *C. neglectus.* Among forest living guenons, *C. hamlyni* and *C. lomamiensis* share semi-terrestrial locomotion with the *lhoesti* species group (*Allochrocebus lhoesti, A. preussi*, and *A. solatus*) and *C. neglectus*. *C. lomamiensis* also shares a similar diet with the *lhoesti* group in which terrestrial herbaceous vegetation is an important component.

Both *C. lomamiensis* and *C. hamlyni* are shy, quiet, and generally occur in small groups, though larger, possibly temporary associations have been reported for *C. hamlyni* [31]. The range of both species is primarily in lowland evergreen tropical forest, although *C. hamlyni* has a larger geographic distribution, occurs across a larger range of altitudes, and utilizes a wider range of habitats, including a variety of montane formations. A similar pattern of speciation in which related species with disjunct ranges and marked genetic and morphological differentiation share common ecological and behavioral traits is seen in the *A. lhoesti* species group. Table 1 provides a summary comparison of distribution, behavior and ecology of *C. lomamiensis* and *C. hamlyni*.

**Table 1:** Comparison of ecology and behavior of *C. lomamiensis* and *C. hamlyni*.

Element	*C. lomamiensis*	*C. hamlyni* ^1^
Known Range	17,000 km^2^	180,000 km^2^
Elevation range	400–615 m	450–3500 m
Habitat	Mature evergreen terra firma forests of the central Lomamiand upper Tshuapa basins including mixed andmonodominant forests.	Mature terra firma and secondary forests from lowlandformations in the west to montane forests and bambooin the Albertine Rift.
Markings	Nose strip diffuse and off-white and blends into paleexposed face skin. Adult male: bright aquamarine scrotumand large perineal patch. Young male: pale gray, or faintlyblue scrotum and perineal patch. Female: pale gray perinealarea, sometimes with a bluish caste.	Nose stripe white, prominent, sharply demarcates the face.Nose stripe reduced or absent in some individuals (see*C. hamlyni kahuziensis*). Adult male: bright aquamarinescrotum and large perineal patch. Variably present blue skinpatches on elbows. Female: No perineal coloration.
Vocal behavior	Low frequency, descending ‘boom’, most frequent duringdawn chorus. Booms can be elicited by imitating eagle calls.	Low frequency, descending ‘boom’, most frequentduring dawn chorus. Booms not readily elicited by imitatingeagle calls.
Olfactory signals	No information	Sternal (apocrine) glands and ritualized chest rubbingobserved in captives.
Positional behavior	Ground to canopy. Feeds, moves and flees on ground.	Ground to canopy. Terrestriality varies by habitat, mostfrequent in montane habitats.
Associations with other primates	Frequent member of multi species associations of primates.	Occasionally joins multi species associations in Ituri Forest.Forages terrestrially with duikers in Ituri.
Group composition and size	Groups of adult females and their offspring and typicallyone adult male. Group size unknown, up to 5 individualsseen together.	Groups of adult females and their offspring and typicallyone adult male. Group size typically 1–15. One group of 22in Nyungwe Forest. One apparently temporary associationof ∼40 individuals seen in the Ituri Forest.
Terrestrial vegetation in diet	Marantaceae important dietary component. Feeds onfallen fruit beneath arboreal primates.	Marantaceae and fungi reported in Ituri. Bamboo in montanehabitats. Feeds on fallen fruit beneath arboreal primates.
Predators	Crowned eagles (*Stephanoaetus coronatus*).	Leopard (*Panthera pardus*) and crowned eagles in Ituri Forest.

**Note:** ^1^ Sources for *C. hamlyni* [31], [43]–[48].

### 2. Biogeography of the *C. hamlyni–C. lomamiensis* Clade

Speciation in African primates has been linked to Pleistocene forest refugia, riverine barriers, and transitional environments [32]–[36]. Pleistocene forest refugia were probably not responsible for the divergence of the *C. hamlyni–C. lomamiensis* clade given the estimated molecular divergence dates near the Pliocene*–*Pleistocene boundary. Instead, biogeographic barriers likely played a role in speciation in the group. *C. lomamiensis* and *C. hamlyni* are separated by two major rivers, the Congo (Lualaba) and the Lomami. Both arise south of the equatorial forest zone and are significant barriers to dispersal of forest faunas. The absence of any member of this clade in the Lomami*–*Lualaba interfluve may reflect inability of these primates to survive in the environments prevalent in much of this region. Particularly in the southern interfluve area, seasonally inundated forests are interspersed with sterile short grass savannas underlain by podzolic soils with a high water table [37], [38]. Other forest taxa, notably okapi (*Okapia johnstoni*) and red colobus (*Procolobus rufomitratus*) with populations east of the Lualaba and west of the Lomami are also absent from these areas of the interfluve region.

### 3. Conservation

*C. lomamiensis* is not uncommon over its range of at least 17,000 km^2^. The species likely remained unknown because the TL2 region (Fig. 1), which includes the lesula’s range, was not well explored biologically until recently. The discovery of *C. lomamiensis* has established the importance of the TL2 region for conservation. Surveys have confirmed and extended preliminary information on the occurrence of a number of forest taxa in the region, notably anthropoid primates. The TL2 region has 11 species or distinctive subspecies of anthropoid primates. Four taxa are endemic to the TL2 region: the lesula, Lomami River red colobus (*Procolobus badius parmentieri*), Lomami River blue monkey (*Cercopithecus mitis heymansi*), and Kasuku River Wolf’s monkey (*Cercopithecus wolfi elegans*) (Table 2).

**Table 2:** Distribution of anthropoid primates of the Tshuapa–Lomami–Lualaba (TL2) region^1^.

Common name 2	Scientific name 2	Distribution within the TL2 region		Endemic to TL2 region
		West Bank Lomami River	East Bank Lomami River to Congo–Lualaba River	
Bonobo	*Pan paniscus*	Present	Present	No
Tshuapa Red Colobus	*Procolobus badius tholloni*	Present	Absent	No
Lomami River Red Colobus	*Procolobus badius parmentieri*	Absent	Present	Yes
Sclater’s Angola Colobus	*Colobus angolensis angolensis*	Present	Present	No
Black Mangabey	*Lophocebus aterrimus*	Present	Present	No
Congo Basin Wolf’s Monkey	*Cercopithecus wolfi wolfi*	Present	Present	No
Kasuku River Wolf’s Monkey	*Cercopithecus wolfi elegans*	Absent	Present	Yes
Lomami River Blue Monkey	*Cercopithecus mitis heymansi*	Present	Present	Yes
Katanga Red-tailed Monkey	*Cercopithecus ascanius katangae*	Present	Present	No
Lesula	*Cercopithecus lomamiensis*	Present	Absent	Yes
De Brazza’s Monkey	*Cercopithecus neglectus*	Present	Present	No

**Notes:** ^1^The TL2 region is approximately delimited on the west by the upper Tshuapa River, on the East by the Lualaba (Congo) River, on the south by the transition from closed forest to savannas, and on the north by the latitude 0.5° S. The Lomami River traverses and bisects the TL2 region from north to south.

2 Taxonomy follows Grubb *et. al.* [49], except for taxonomy of red colobus, which follows Ting [50].

At present, TL2 remains remote from human expansion, and there is no logging or mining. Hunting is the immediate threat to the faunas of TL2. We provide a provisional IUCN Red List Assessment of Vulnerable [39] for *C. lomamiensis* based on the inferred population decline from uncontrolled bushmeat hunting. This has expanded into the species’ range over the last decade driven by the urban bushmeat markets of Kindu and Kisangani [40]. For species with restricted ranges and reliance on primary forest, such as *C. lomamiensis*, uncontrolled hunting can lead to catastrophic declines over a short period [41].

The conservation of *C. lomamiensis* urgently requires controls on hunting along with the creation of a protected area. A core zone of 9000 km^2^ in the TL2 region is proposed as the Lomami National Park (see [42] for documentation of the gazettement process). The proposed protected area, along with the existing Réserve Naturelle de Sankuru, will cover most of the known range of *C. lomamiensis*. While the establishment of the new protected area is a necessary first step, active protection and monitoring are required to ensure the conservation of the lesula and other unique biodiversity of the eastern rim of Congo’s central basin.

## Supporting Information

Figure S1 **Maximum likelihood tree, TSPY.** Phylogram and bootstrap support values (500 replicates) were inferred using GARLI 0.951. The topology is identical to that inferred using a Bayesian approach (Fig. S2). *Cercopithecus lomamiensis* and *C. hamlyni* are reciprocally monophyletic. The scale at the bottom is in units of nucleotide substitutions per site.
(PDF)

Figure S2 **Bayesian tree, TSPY.** Phylogram and clade credibility scores were obtained using MRBAYES 3.11. The topology is identical to the ML tree. The scale at the bottom is in units of nucleotide substitutions per site.
(PDF)

Figure S3 **Maximum likelihood tree, Xq13.3 homolog.** The scale at the bottom is in units of nucleotide substitutions per site.
(PDF)

Figure S4 **Bayesian tree, Xq13.3 homolog.** The scale at the bottom is in units of nucleotide substitutions per site.
(PDF)

Table S1 **Specimens of** ***Cercopithecus lomamiensis*** **and** ***Cercopithecus hamlyni*** **examined for this study.**
(PDF)

Table S2 **Genetic samples list.**
(PDF)

Table S3 **Selected cranial measurements of** ***Cercopithecus lomamiensis*** **(to the nearest tenth of a millimeter).**
(PDF)

Table S4 **Comparative craniodental measurements between** ***Cercopithecus lomamiensis*** **and** ***Cercopithecus hamlyni*** **.**
(PDF)

Table S5 **Cranial landmarks used in the 3-dimensional comparative analysis of cranial morphology.**
(PDF)

Table S6 **Acoustic parameters measured for each sampled boom.**
(PDF)

Table S7 **Brief descriptions of** ***Cercopithecus lomamiensis*** **crania examined (by specimen).**
(PDF)

Table S8 **Parameters of boom calls of** ***Cercopithecus hamlyni*** **and** ***Cercopithecus lomamiensis*** **.**
(PDF)

Table S9 **Estimated divergence dates (million years ago) in Cercopithecid lineages A) TSPY, B) Xq13.3 homolog.** Constrained nodes are marked with *.
(PDF)

Text S1 **Information and references on divergence date calibration methods.**
(PDF)

Audio S1 **Exemplars of booms by** ***Cercopithecus hamlyni*** **.**
(AIF)

Audio S2 **Exemplars of booms by** ***Cercopithecus lomamiensis*** **.**
(AIF)

Matrix S1 **DNA sequence alignment for TSPY.**
(NEX)

Matrix S2 **DNA sequence alignment for the Xq13.3 homolog.**
(NEX)
