# Supplementary material for: Lesula: A New Species of Cercopithecus Monkey Endemic to the Democratic Republic of Congo and Implications for Conservation of Congo’s Central Basin
Source: PLoS One. 2012 Sep 12;7(9):e44271. doi: 10.1371/journal.pone.0044271 (PMC3440422; doi:10.1371/journal.pone.0044271)
Supplement: Figure S2 — Bayesian tree, TSPY. Phylogram and clade credibility scores were obtained using MRBAYES 3.11. The topology is identical to the ML tree. The scale at the bottom is in units of nucleotide substitutions per site. (PDF) [file pone.0044271.s002.pdf]

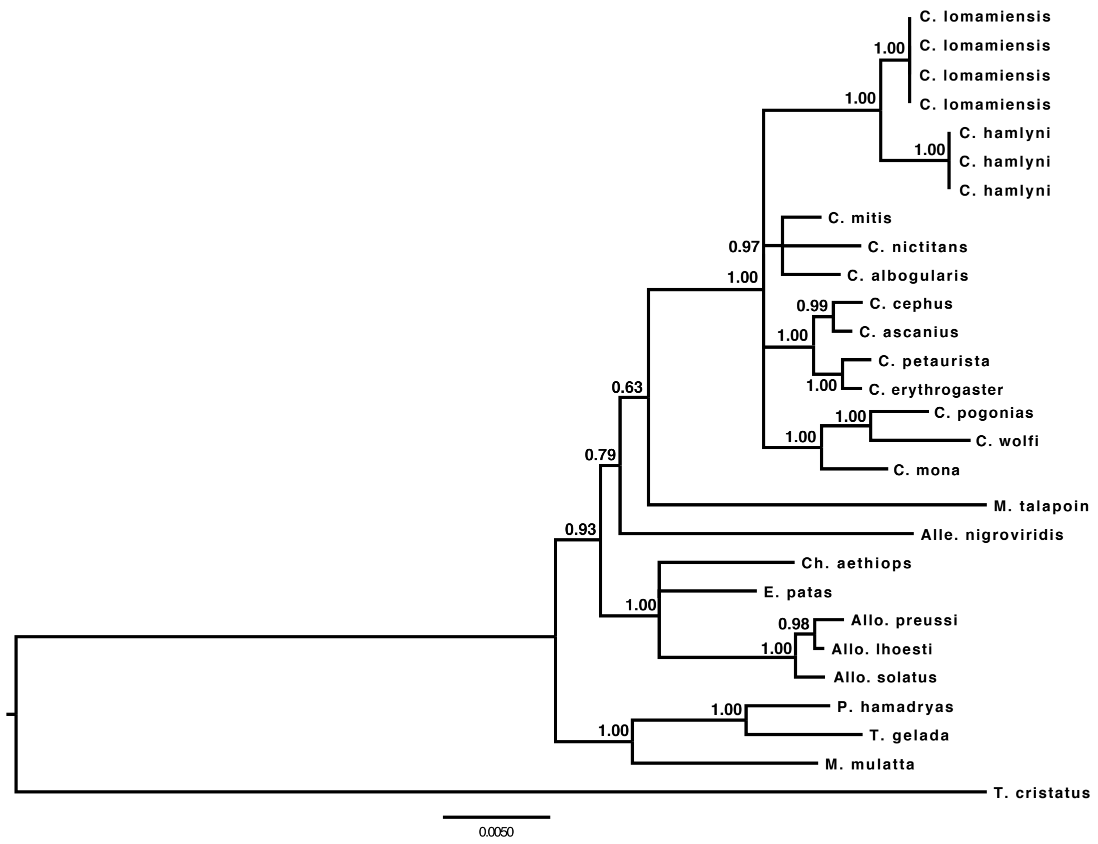

Fig. S2. Bayesian tree, TSPY. Phylogram and clade credibility scores were obtained using MRBAYES 3.11. The topology is identical to the ML tree. The scale at the bottom is in units of nucleotide substitutions per site.
