# Supplementary material for: Lesula: A New Species of Cercopithecus Monkey Endemic to the Democratic Republic of Congo and Implications for Conservation of Congo’s Central Basin
Source: PLoS One. 2012 Sep 12;7(9):e44271. doi: 10.1371/journal.pone.0044271 (PMC3440422; doi:10.1371/journal.pone.0044271)
Supplement: Figure S3 — Maximum likelihood tree, Xq13.3 homolog. The scale at the bottom is in units of nucleotide substitutions per site. (PDF) [file pone.0044271.s003.pdf]

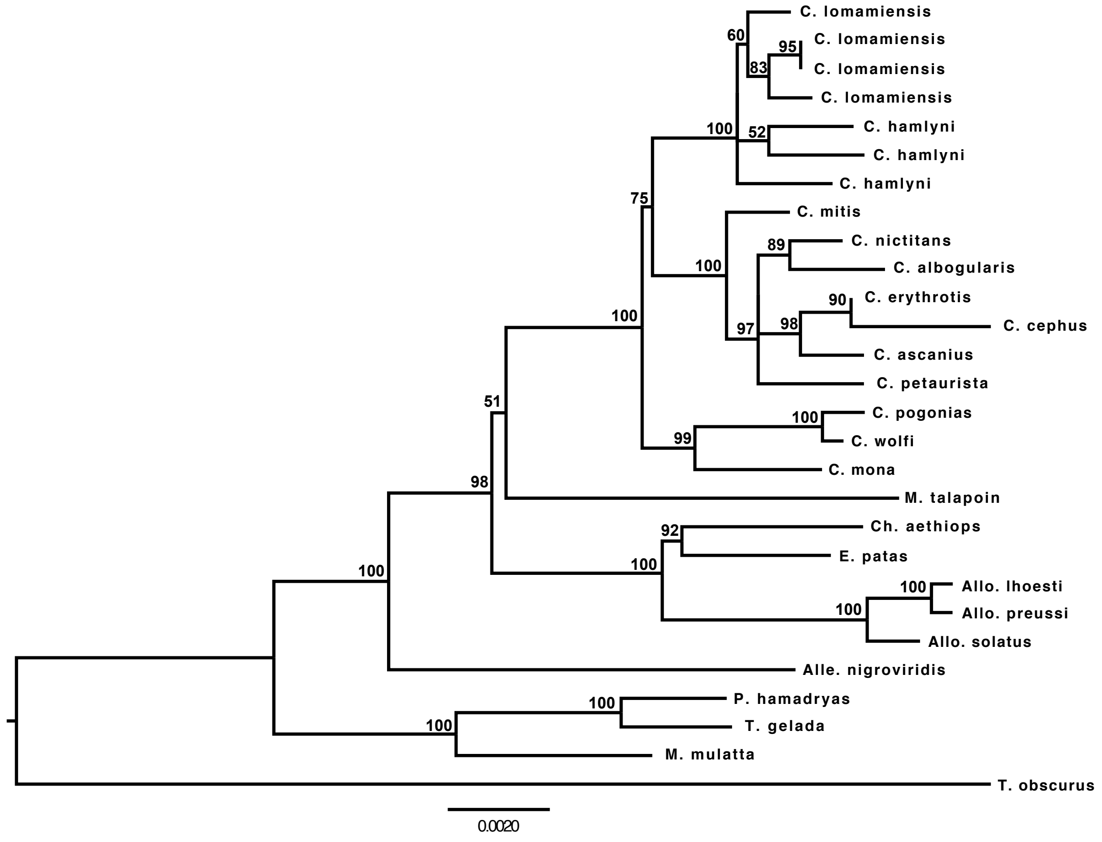

Fig. S3. Maximum likelihood tree, Xq13.3 homolog. The scale at the bottom is in units of nucleotide substitutions per site.
