# Supplementary material for: Lesula: A New Species of Cercopithecus Monkey Endemic to the Democratic Republic of Congo and Implications for Conservation of Congo’s Central Basin
Source: PLoS One. 2012 Sep 12;7(9):e44271. doi: 10.1371/journal.pone.0044271 (PMC3440422; doi:10.1371/journal.pone.0044271)
Supplement: Table S1 — Specimens of Cercopithecus lomamiensis and Cercopithecus hamlyni examined for this study. (PDF) [file pone.0044271.s005.pdf]

Table S1. Specimens of *Cercopithecus lomamiensis* and *Cercopithecus hamlyni* examined for this study.

Specimen ID	Species	Sex	Age	Provenance of specimen		Standard measures (cm)					Material analyzed			
				Latitude	Longitude	Total length	Tail	Hind foot	Ear	Mass (kg)	Skin	Skull	Tissue	Provenance and note
YPM 14080	*lomamiensis*	M	Adult	S1.02237	E24.42368	125	65	14.0	4.0	4.0	+	+	+	Hunter kill, Type specimen
YPM 14189	*lomamiensis*	F	Subadult	S1.06571	E24.44838	105	62.5	13.4	4.2	3.5	+	+		Hunter kill
YPM 14190	*lomamiensis*	F	Subadult	S1.42801	E25.01601	110	60	13.5	2.8		+	+		Hunter kill
YPM 14191	*lomamiensis*	M	Adult	S1.38145	E25.03843	125	78	16.5	3.4	7.1	+	+	+	Hunter kill
YPM 14192	*lomamiensis*	F	Subadult	S1.40129	E24.97498	87	47	13.5	3.2	4.0	+	+		Eagle kill
GP 600	*lomamiensis*	M	Juvenile	S1.06571	E24.44838								+	Captive
JH 005	*lomamiensis*	M	Adult	S1.50401	E24.93501						+		+	Hunter kill
YPM 14193	*hamlyni*	M	Subadult	N1.34005	E28.65038							+		Leopard kill
YPM 14194	*hamlyni*	M	Adult	N1.34005	E28.65038							+		
YPM 14195	*hamlyni*	M	Adult	N1.34005	E28.65038							+		Leopard kill
AMNH90028	*hamlyni*	M	Adult	S2.31716	E28.73163						+			Mt. Kahuzi
YPM 17272	*hamlyni*	M	Adult	N0.77249	E25.25894	123	75	10.2	3.8	12.5		+	+	
ME 404	*hamlyni*	M	Adult	N0.77249	E25.25894								+	
AMNH 86948	*hamlyni*	M	Adult	S1.48988	E29.43844							+		Mt. Karisimbi
AMNH 81000	*hamlyni*	F	Adult	S1.95496	E28.86263							+		Lake Kivu region

Notes: Abbreviations are as follows: YPM = Yale Peabody Museum; AMNH = American Museum of Natural History; GP = Gilbert Paluku, collector; JH = John Hart, collector; ME = Maurice Emetshu, collector. Empty cells = data unavailable.
