# Supplementary material for: Lesula: A New Species of Cercopithecus Monkey Endemic to the Democratic Republic of Congo and Implications for Conservation of Congo’s Central Basin
Source: PLoS One. 2012 Sep 12;7(9):e44271. doi: 10.1371/journal.pone.0044271 (PMC3440422; doi:10.1371/journal.pone.0044271)
Supplement: Table S2 — Genetic samples list. (PDF) [file pone.0044271.s006.pdf]

Table S2. Genetic samples list.

Taxon	Origin	Sample ID	Source	GenBank # (TSPY, Xq13.3 homolog)
Chlorocebus aethiops	East Africa	90.017	Dept. Anthropology, NYU	AY450873, AY899216
Cercopithecus albogularis	Africa	89M009	Cheyenne Mountain Zoo	EF517803, AY899220
Cercopithecus ascanius	Central Africa	41137B	Louisiana Purchase Zoo	EF517804, AY899222
Cercopithecus cephus	Central Africa	The.Zoo.1	Gulf Breeze Zoo	AY450874, AY899223
Cercopithecus erythrogaster[a]	West Africa	Mulhouse3	Mulhouse Zoo	AY665648, -------------
Cercopithecus erythrotis[b]	Bioko, Equatorial Guinea	066-B	CIRMF, Gabon	-------------, AY899225
Cercopithecus hamlyni	Central Africa	OR.1646	San Diego Zoo	AY450875, AY899231
Cercopithecus hamlyni	Eastern Democratic Republic of the Congo (DRC)	ME404	Drs. J. and T. Hart, TL2	JN106053, JN106059
Cercopithecus hamlyni	Eastern DRC	GP612	Drs. J. and T. Hart, TL2	JN106052, JN106058
Allochrocebus lhoesti	Central Africa	Antwerp	Antwerp Zoo	AY048055, AY899217
Cercopithecus lomamiensis	Central Congo Basin, DRC	GP600	Drs. J. and T. Hart, TL2	JN106054, JN106062
Cercopithecus lomamiensis	Central Congo Basin, DRC	GP609	Drs. J. and T. Hart, TL2	JN106055, JN106063
Cercopithecus lomamiensis	Central Congo Basin, DRC	JH005	Drs. J. and T. Hart, TL2	JN106056, JN106061
Cercopithecus lomamiensis	Central Congo Basin, DRC	ME408	Drs. J. and T. Hart, TL2	JN106057, JN106060
Cercopithecus mitis	Africa	C.Lehn	Dr. C. Lehn	AY450876, AY899219
Cercopithecus mona	Grenada	SA.BF#3	Dr. M. Glenn/K. Bensen	AF284281, AY899227
Cercopithecus nictitans	Central Africa	Newton	BREC's Baton Rouge Zoo	AY450878, AY899221

Table S2. continued.

**Taxon**	**Origin**	**Sample ID**	**Source**	**GenBank # (TSPY, Xq13.3 homolog)**
Cercopithecus petaurista	West Africa	1534	Central Florida Zoo	AY897616, AY899224
Cercopithecus pogonias	Gabon	108-G	CIRMF, Gabon	EF517805, AY899229
Allochrocebus preussi	Bioko, Equatorial Guinea	008-B	Dr. P. Telfer, WCS	EF517802, EF517809
Allochrocebus solatus	Gabon	Omboue	CIRMF, Gabon	AY450879, AY899218
Cercopithecus wolfi	Central Africa	Nguma	San Antonio Zoo	AY450880, AY899228
Allenopithecus nigroviridis	Congo	R146/97	National Museum, Scotland	AF284280, AY899233
Erythrocebus patas	Africa	R230, R229	Dept. Anthropology, CUNY	AY048064, AY899215
Miopithecus talapoin	Central Africa	1511	Audubon Zoo	EF517806, AY899232
Macaca mulatta	China	20156, 19874	CERC, Columbia University	AF284259, AY899239
Papio hamadryas	Africa	73.347, 18742	NYU/Southwest Foundation	AF284277, AY899234
Theropithecus gelada	Ethiopia	891096	Dept. Anthropology, NYU	AF284278, AY899236
Trachypithecus spp.	Southeast Asia	DJ.1, CBS	Drs. D. Melnick/C.-B. Stewart	AF284232, EF517811

Notes: [a] We did not have a *Cercopithecus erythrogaster* sample for this study. We included a TSPY sequence available on GenBank, but no Xq13 sequence exists for this taxon. [b] Our *Cercopithecus erythrotis* sample is from a female, and therefore does not carry a Y-chromosome.
