# Supplementary material for: Lesula: A New Species of Cercopithecus Monkey Endemic to the Democratic Republic of Congo and Implications for Conservation of Congo’s Central Basin
Source: PLoS One. 2012 Sep 12;7(9):e44271. doi: 10.1371/journal.pone.0044271 (PMC3440422; doi:10.1371/journal.pone.0044271)
Supplement: Table S3 — Selected cranial measurements of Cercopithecus lomamiensis (to the nearest tenth of a millimeter). (PDF) [file pone.0044271.s007.pdf]

Table S3. Selected cranial measurements of *Cercopithecus lomamiensis* (to the nearest tenth of a millimeter).

Specimen ID	Age/Sex	Pr-In	Pr-Ba	Na-Ba	Gl-In	Br-Ba	Min. Frontal Breadth	Po-Po	Gl-Br	La-In	Op-In	Na-Pr	Na-Rh	Zy-Zy	Max Orbital Height	Max Orbital Width
YPM 14080	Adult male	113.4	78.2	59.1	79.2	51.5	45.7	59.3	44.6	17	23.5	54	26.6	71	25.6	24.7
YPM 14189	Subadul female	97.1	66.6	54	72	48.8	44.8	54.2	41.9	16.2	20.1	42.5	22.8	61.4	23.9	22.8
YPM 14190	Subadult female	91.0 (estimate)	X	X	68.0 (estimate)	X	42.7	50.1	39.3	X	X	34.3	16.1	55.3	23.1	20.7
YPM 14191	Adult male	116.4	80.5	58.8	80.7	54.8	47.2	60.9	42.8	12.1	24.4	52.6	22.3	71.4	22.9	24.9
YPM 14192	Subadult female	94.5	62.6	51.9	71.8	48.2	43.6	54.3	40.8	16.3	20.1	41.1	20.2	58.7	21.3	22.5

Notes: Pr=Prosthion, In=Inion, Ba=Basion, Na=Nasion, Gl=Glabella, Br=Bregma, Po=Porion, La=Lambda, Op=Opisthion, Rh=Rhinion, Zy=Zygion. X = Measurement unavailable.
