# Supplementary material for: Lesula: A New Species of Cercopithecus Monkey Endemic to the Democratic Republic of Congo and Implications for Conservation of Congo’s Central Basin
Source: PLoS One. 2012 Sep 12;7(9):e44271. doi: 10.1371/journal.pone.0044271 (PMC3440422; doi:10.1371/journal.pone.0044271)
Supplement: Table S4 — Comparative craniodental measurements between Cercopithecus lomamiensis and Cercopithecus hamlyni . (PDF) [file pone.0044271.s008.pdf]

Table S4. Comparative craniodental measurements between *Cercopithecus lomamiensis* and *Cercopithecus hamlyni*.

	***C. lomamiensis* (Pooled)**				***C. hamlyni* (Pooled)**				**Significance**
**Character**	**Mean (mm)**	**Range (mm)**	**Size-adjusted Mean**	**n**	**Mean (mm)**	**Range (mm)**	**Size-adjusted Mean**	**n**	**p-value**
Posterior occipital length (lambda-inion)	15.1	12.1-17.0	0.686	3	11.1	9.2 - 12.8	0.510	6	0.044*
Calvarial shape (biporionic breadth / glabella-inion)	0.752	0.749 - 0.755	0.752	3	0.792	0.756 - 0.823	0.792	6	0.015*
Inferior occipital length (opisthion-inion)	22.7	20.1 - 24.4	1.02	3	24.3	21.7 - 26.2	1.12	5	0.024*
Anterior interorbital breadth (bimaxilliofrontale)	6.3	4.3 - 7.3	0.283	3	7.6	6.1 - 8.7	0.348	6	0.039*
Orbit area (max orbit width x max orbit height)	582.5	544.9 - 632.3	26.2	3	488.2	434.4 - 535.6	22.4	7	0.023*
Occipital flexion	150.3	142.9 - 155.9	150.3	3	138.5	134.5 - 151.7	138.5	7	0.087
I^1 area (MD length x BL width)	25.8	25.3 - 26.3	1.10	2	18.2	14.6 - 20.9	0.843	5	0.025*
I^2 area (MD length x BL width)	13.7	11.9 - 15.5	0.58	2	9.5	9.0 - 9.9	0.43	6	0.273
M^2 area (MD length x BL width)	53.8	52.6 - 55.4	2.43	3	41.7	34.8 - 46.9	1.90	7	0.002*
M^3 area (MD length x BL width)	42.6	41.6 - 43.6	1.81	2	29.9	26.0 - 34.8	1.35	6	0.000*
I_1 area (MD length x BL width)	13.1	10.8 - 14.8	0.592	3	9.9	7.0 - 12.2	0.452	7	0.053
I_2 area (MD length x BL width)	13.0	9.5 - 15.1	0.576	3	10.0	8.4 - 12.4	0.454	5	0.068
M_2 area (MD length x BL width)	45.9	42.9 - 50.8	2.07	3	36.8	29.4 - 44.1	1.68	7	0.021*

Table S4. continued.

	C. lomamiensis (Males only)				*C. hamlyni* (Males only)				Significance
**Character**	**Mean (mm)**	**Range (mm)**	**Size-adjusted Mean**	**n**	**Mean (mm)**	**Range (mm)**	**Size-adjusted Mean**	**n**	**p-value**
Calvarial shape (biporionic breadth / glabella-inion)	0.752	0.749 - 0.755	0.752	2	0.798	0.756 - 0.823	0.798	5	0.059
Max cranial width (bieuryonic breadth)	61.2	60.8 - 61.6	2.60	2	59.6	56.4 - 64.1	2.74	5	0.025*
Orbit area (max orbit width x max orbit height)	601.3	570.2 - 632.3	25.55	2	487.79	434.4 - 535.6	22.08	6	0.088
I^1 area (MD length x BL width)	25.8	25.3 - 26.3	1.10	2	19.1	17.2 - 20.9	0.875	4	0.015*
I^2 area (MD length x BL width)	13.7	11.9 - 15.5	0.581	2	9.5	9.0 -9.9	0.425	5	0.264
M^2 area (MD length x BL width)	54.0	52.6 - 55.4	2.29	2	42.8	39.7 - 46.9	1.94	6	0.002*
M^3 area (MD length x BL width)	42.6	41.6 - 43.6	1.81	2	30.6	27.0 - 34.8	1.37	5	0.002*

Notes: * = Significant difference at the 0.05 level. Pooled = males and females included together in the analysis. Occipital flexion = angle between opisthion-basion and opisthion-inion. MD = mesiodistal, BL = buccolingual. In order to maximize sample sizes, specimens were included in the analysis if the M3/m3s had begun eruption. Size-adjusted values were obtained by dividing the raw measurements for each specimen by a geometric mean of 41 cranial measurements for each specimen, where applicable. T-tests were performed using the size-adjusted values for each character. *C. hamlyni* specimens analyzed include YPM 17272, YPM 14195, YPM 14194, YPM 14193, AMNH 86948, AMNH 90028, AMNH 81000. *C. lomamiensis* specimens analyzed include YPM 14189, YPM 14080, YPM 14191.
