# Supplementary material for: Lesula: A New Species of Cercopithecus Monkey Endemic to the Democratic Republic of Congo and Implications for Conservation of Congo’s Central Basin
Source: PLoS One. 2012 Sep 12;7(9):e44271. doi: 10.1371/journal.pone.0044271 (PMC3440422; doi:10.1371/journal.pone.0044271)
Supplement: Table S5 — Cranial landmarks used in the 3-dimensional comparative analysis of cranial morphology. (PDF) [file pone.0044271.s009.pdf]

Table S5. Cranial landmarks used in the 3-dimensional comparative analysis of cranial morphology.

Landmark Code	Description
**1**	Rhinion
**2**	Nasion
**3**	Postorbital sulcus/depression in the midline
**4**	Bregma
**5**	Medial orbit border
**6**	Lateral orbit border
**7**	Orbitale superior
**8**	Orbitale inferior
**9***	"Pterion" (most anterior point on the braincase in the infratemporal fossa)
**10***	Zygion
**11***	Ectomolare
**12***	Euryon
**13***	Prosthion
**14**	Lambda
**15**	Inion
**16**	Opisthion
**17**	Basion
**18**	Sphenobasion
**19**	Alveolon
**20**	Porion
**21**	Petrous apex
**22**	Carotid canal
**23**	Most anterior point on jugular foramen

Notes: * Indicates landmark collected in both superior and inferior views. The two sets of measurements were then combined using the program DVLR v. 0.4.9 [15] to obtain a single set of 23 three-dimensional coordinates for each cranium. Only individuals acquired in the wild were included in the analysis. *Cercopithecus hamlyni* specimens included: YPM 17272, YPM 14195, YPM 14194, YPM 14193, AMNH 86948, AMNH 90028, AMNH 81000. *Cercopithecus lomamiensis* specimens included: YPM 14189, YPM 14080, YPM 14191.
