# Supplementary material for: Lesula: A New Species of Cercopithecus Monkey Endemic to the Democratic Republic of Congo and Implications for Conservation of Congo’s Central Basin
Source: PLoS One. 2012 Sep 12;7(9):e44271. doi: 10.1371/journal.pone.0044271 (PMC3440422; doi:10.1371/journal.pone.0044271)
Supplement: Table S6 — Acoustic parameters measured for each sampled boom. (PDF) [file pone.0044271.s010.pdf]

Table S6. Acoustic parameters measured for each sampled boom.

PARAMETER	DESCRIPTION
Start Freq (Hz)	Peak frequency during first 10% of signal.
End Freq (Hz)	Peak frequency during last 10% of signal.
Low freq (Hz)	Lowest frequency in the signal.
High freq (Hz)	Highest frequency in the signal.
Q1 freq (Hz)	Peak frequency at time when 25% of signal's energy had past (i.e. at end of the first quarter).
Q3 Freq (Hz)	Peak frequency at time when 75% of signal's energy had past (i.e. at end of the third quarter).
Center Freq (Hz)	The frequency dividing signal into 2 frequency intervals of equal energy
IQR bandwidth (Hz)	Inter-quartile range bandwidth.
IQR Dur (secs)	Inter-quartile range duration: difference between 1st and 3rd quartile times. Provides duration of interval in which 50% of call's energy occurred.
Max Freq (Hz)	The frequency at which maximum power (amplitude) of call occurs.
Duration (secs)	Duration of entire signal.
*Slope: Total (Hz/sec)	(End Freq–Start Freq) / Duration
*Slope: 1st quart (Hz/sec)	(Q1 freq–Start Freq) / duration of first quarter
*Slope: Middle (Hz/sec)	(Q3 freq–Q1 Freq) / duration of middle half
*Slope: 4th quart (Hz/sec)	(End freq–Q3 Freq) / duration of last quarter
*Delta Freq (Hz)	Highest frequency minus lowest frequency.

Note: * Indicates measure derived from other parameters.
