# Supplementary material for: Lesula: A New Species of Cercopithecus Monkey Endemic to the Democratic Republic of Congo and Implications for Conservation of Congo’s Central Basin
Source: PLoS One. 2012 Sep 12;7(9):e44271. doi: 10.1371/journal.pone.0044271 (PMC3440422; doi:10.1371/journal.pone.0044271)
Supplement: Table S7 — Brief descriptions of Cercopithecus lomamiensis crania examined (by specimen). (PDF) [file pone.0044271.s011.pdf]

Table S7. Brief descriptions of *Cercopithecus lomamiensis* crania examined (by specimen).

Specimen ID	Age sex	Description
YPM 14080	Adult male	Complete cranium with mandible, all permanent molars are present, upper incisors and right P3s are present, but loose from the specimen. The lower canines appear to be fully erupted, broken, and worn. The upper canines are ~90% emerged. All sutures/synchondroses are fused.
YPM 14189	Subadult female	Complete cranium with mandible, premolars, M1/m1s, and M2/m2s are present, M3/m3s just beginning eruption. Canines appear erupted. Upper incisors and lower left lateral incisor are missing. Spheno-occipital synchondrosis is not yet fully fused.
YPM 14190	Subadult female	Mostly complete cranium with mandible. Occipital is missing from skull. M1/m1s are present, M2/m2s are just beginning eruption, dP3/dp3 and dP4/dp4s are still present.
YPM 14191	Adult male	Complete cranium with mandible. Cranium is damaged (right parietal is broken inwards); all permanent molars and premolars are present. The upper and lower canines are ~90% erupted. Spheno-occipital synchondrosis is not yet fully fused.
YPM 14192	Subadult female	Complete cranium with mandible. M1/m1s and M2/m2s are present. Upper premolars are present, lower p4 is present, lower p3 was most likely present, but only the alveolus remains. Upper incisors and canines are missing, and the lower incisors, canines and p3s are also missing. M3/m3s have not yet begun eruption. Spheno-occipital synchondrosis is not yet fully fused.
