# Supplementary material for: Lesula: A New Species of Cercopithecus Monkey Endemic to the Democratic Republic of Congo and Implications for Conservation of Congo’s Central Basin
Source: PLoS One. 2012 Sep 12;7(9):e44271. doi: 10.1371/journal.pone.0044271 (PMC3440422; doi:10.1371/journal.pone.0044271)
Supplement: Table S8 — Parameters of boom calls of Cercopithecus hamlyni and Cercopithecus lomamiensis . (PDF) [file pone.0044271.s012.pdf]

Table S8. Parameters of boom calls of *Cercopithecus hamlyni* and *Cercopithecus lomamiensis*.

	C. hamlyni **(n=43)**		***C. lomamiensis*** **(n=6)**		**t-test**	
**PARAMETER**	**Mean ±SD**	**Range**	**Mean ±SD**	**Range**	**t**	**p**
Start Freq (Hz)	204.8±17.5	172 – 247	215±9.8	204 – 226	-2.11	0.061
End Freq (Hz)	188.4±14.9	161 – 215	197.8±8.9	193 – 215	-2.90	0.054
Low freq (Hz)	173.6±16.3	147 – 210	181.9±10.4	172 – 201	-1.68	0.127
High freq (Hz)	220.8±16.9	189 – 162	234.3±9.7	220 – 246	-2.85	0.017^{s}
Q1 freq (Hz)	190.0±16.1	162 – 226	199.2±9.0	194 – 215	-2.07	0.065
Q3 Freq (Hz)	204.6±15.4	172 – 237	213.5±8.1	205 – 226	-2.21	0.049^{s}
Center Freq (Hz)	197.1±15.3	172 – 237	204.6±9.6	194 – 215	-1.64	0.134
IQR bandwidth (Hz)	14.5±5.7	10.8 – 32.3	14.4±5.5	11 – 22	0.07	0.946
IQR Dur (secs)	0.15±0.05	0.1 – 0.2	0.13±0.05	0.1 – 0.2	0.69	0.514
Max Freq (Hz)	198.3±15.5	172 – 237	204.6±6.8	194 – 215	-1.71	0.109
Duration (secs)	0.37±0.07	0.25 – 0.49	0.34±0.06	0.22 – 0.39	1.24	0.254
Slope: Total (Hz/sec)	-45.0±22.7	-98.5 – 0.0	-52.3±28.12	-105 – -29	0.61	0.563
Slope: 1st quart (Hz/sec)	-166.2±112.3	-646.9 – -53.5	-179.9±78.8	-315 – -91.5	0.38	0.717
Slope: Middle (Hz/sec)	101.5±54.2	36 – 216	87.2±37.6	54 – 135	0.82	0.437
Slope: 4th quart (Hz/sec)	-205.3±164.4	-921 – 0	-306.5±185.1	-528 – -105	1.27	0.250
Delta Freq (Hz)	47.2±9.7	35.3 – 77.8	52.4±9.37	39.5 – 65.4	-1.28	0.245

Note: s = significant difference ($p<0.05$).
