# Supplementary material for: Lesula: A New Species of Cercopithecus Monkey Endemic to the Democratic Republic of Congo and Implications for Conservation of Congo’s Central Basin
Source: PLoS One. 2012 Sep 12;7(9):e44271. doi: 10.1371/journal.pone.0044271 (PMC3440422; doi:10.1371/journal.pone.0044271)
Supplement: Table S9 — Estimated divergence dates (million years ago) in Cercopithecid lineages A) TSPY, B) Xq13.3 homolog. Constrained nodes are marked with *. (PDF) [file pone.0044271.s013.pdf]

Table S9. Estimated divergence dates (million years ago) in Cercopithecid lineages A) TSPY, B) Xq13.3 homolog. Constrained nodes are marked with *.

**A. TSPY**		**95% confidence intervals**	
SPLIT	MEAN	MAX	MIN
Colobinae – Cercopithecinae *	15.6	20.3	10.9
Papionini – Cercopithecini	9.9	14.2	7.1
Macaca – Afro-papionins *	7.7	9.7	5.8
Papio – Theropithecus *	4.7	5.5	4.0
terrestrials – rest of Cercopithecini	7.6	12.6	5.1
vervet – patas – *lhoesti* group	5.0	8.4	2.0
lhoesti group MRCA	1.2	2.3	0.2
Allochrocebus lhoesti – Allo. preussi	0.5	1.1	0.1
Allenopithecus – Miopithecus/arboreals	7.2	11.7	4.5
Miopithecus – arboreals	6.8	10.7	3.8
hamlyni group – *mitis* group – *cephus* group – *mona* group	4.3	7.3	2.2
mitis group MRCA	2.2	4.4	0.8
cephus group MRCA	2.0	3.2	0.5
Cercopithecus cephus – C. ascanius	1.1	1.7	0.2
C .petaurista – C. erythrogaster	0.9	1.6	0.2
mona group MRCA	2.4	4.6	1.1
C. wolfi – C. pogonias	1.6	2.9	0.5
hamlyni group MRCA	1.7	3.2	0.5
C. lomamiensis MRCA	0.5	1.1	0.1
C. hamlyni MRCA	0.4	0.7	0.1
**B. Xq13.3 homolog**			
SPLIT	MEAN	MAX	MIN
Colobinae – Cercopithecinae *	16.5	20.8	12.2
Papionini – Cercopithecini	12.2	17.3	9.8
Macaca – Afro-papionins *	8.0	9.8	6.3
Papio – Theropithecus *	4.6	5.2	4.0
Allenopithecus – rest of Cercopithecini	9.9	15.6	7.6
terrestrials – *Miopithecus*/arboreals	8.8	13.0	6.1
terrestrials MRCA	5.5	7.9	3.2
vervet – patas	4.1	6.7	2.3
lhoesti group MRCA	1.5	3.1	0.7
Allochrocebus lhoesti – Allo. preussi	0.6	1.2	0.2
Miopithecus – arboreals	7.8	12.1	5.5
arboreals MRCA	4.9	8.1	3.3
mona group – *cephus/mitis/hamlyni* groups	4.8	7.5	3.3
mona group MRCA	3.1	6.1	2.1
Cercopithecus wolfi – C. pogonias	0.7	1.7	0.3
hamlyni group – *mitis/cephus* group	4.8	7.4	3.3
C. mitis – C. ascanius/C. cephus/C. erythrotis/C. nictitans/C. albogularis/ C. petaurista	3.4	5.2	2.1
C .ascanius/C. cephus/C. erythrotis – C. nictitans/C. albogularis – C. petaurista	2.9	4.3	1.6

Table S9. continued.

**B. Xq13.3 homolog**		**95% confidence intervals**	
SPLIT	MEAN	MAX	MIN
C. nictitans – C. albogularis	1.8	2.9	0.8
C. ascanius – C. cephus/C. erythrotis	2.0	3.2	1.1
C. ascanius – C. cephus/C. erythrotis	2.0	3.2	1.1
C. erythrotis – C. cephus	1.4	2.0	0.5
hamlyni group MRCA	2.8	4.3	1.6
C. lomamiensis MRCA	1.9	4.0	0.8
