# Supplementary material for: Lesula: A New Species of Cercopithecus Monkey Endemic to the Democratic Republic of Congo and Implications for Conservation of Congo’s Central Basin
Source: PLoS One. 2012 Sep 12;7(9):e44271. doi: 10.1371/journal.pone.0044271 (PMC3440422; doi:10.1371/journal.pone.0044271)
Supplement: Text S1 — Information and references on divergence date calibration methods. (PDF) [file pone.0044271.s014.pdf]

Supporting Information Text: Information and references on divergence date calibration methods.

Most estimates of cercopithecid divergence dates use several standard calibration points, including hominoid-cercopithecoid split and the divergence between *Pan* and *Homo*, which are supported by fossil evidence. These studies also root their datasets with primates other than hominoids (typically platyrrhines), as hominoids display a significantly slower rate of molecular evolution relative to other anthropoids [e.g., S1]. However, we could not take this approach because clearly orthologous hominoid or platyrrhine sequences were not available on GenBank for both loci. We therefore constrained our datasets using three calibration points: 1) the colobine / cercopithecine split; 2) the divergence between *Macaca* and the extant Afro-papionins (*Cercocebus*, *Mandrillus*, *Lophocebus*, *Rungwecebus*, *Theropithecus*, and *Papio*); and 3) the *Papio* / *Theropithecus* split. As the fossil record of the Cercopithecini is poor, our calibration points had to come from outside that group.

Colobinae / Cercopithecinae

The earliest fossil colobines, *Mesopithecus* and *Microcolobus*, are present from ~10 million years ago (Ma), while the first known cercopithecines appear somewhat later [S2, S3]. However, the two groups probably arose from a common ancestor in Africa, and the fossil record in Africa is poor for the period 14–8 Ma. As a result, there is some uncertainty about how much earlier than 10 Ma the two would have diverged. In the period 19–12.5 Ma, fossils belonging to the stem cercopithecoid family Victoriapithecidae are relatively common [S2, S4]. Previous molecular divergence date estimates suggest that cercopithecines and colobines diverged between 20–13 Ma [S5–

S11]. Given these data, this node was rather broadly constrained using three different prior distributions of the Time to Most Recent Common Ancestor (TMRCA) of the Colobinae / Cercopithecinae: a uniform distribution of 19–11 Ma, a normal distribution with a mean of 15 Ma and a standard deviation of 2.5 (95% interval = 19.1–10.9 Ma), and a lognormal distribution with log(mean) of 1.5, log(standard deviation) of 0.5, and an offset of 9 (95% interval = 19.2–10.9 Ma, median = 13.5 Ma). The three prior distributions resulted in very similar date estimates across all nodes of the tree.

Macaca / extant Afro-papionins

Macaca first appears around 5.5 Ma, and fossil and paleobiogeographic data suggest that *Macaca* and the living Afro-papionins diverged between 10–7 Ma [S2]. Molecular estimates are in concordance with this, estimating the divergence to have occurred somewhere in the period of 10 Ma to 6 Ma [S5, S7, S12]. This node was consequently constrained with three different prior distributions of the *Macaca* / Afro-papionin TMRCA: a uniform distribution of 10–6 Ma, a normal distribution with a mean of 8 Ma and a standard deviation of 1.25 (95% interval = 10.1–5.9 Ma), and a lognormal distribution with log(mean) = 0.8, log(standard deviation) = 0.5, and an offset of 5 (95% interval = 10.1–6 Ma, median = 7.2 Ma). These different prior distributions also resulted in very similar date estimates across the tree. The dates displayed in Figures 9 and 10 of the main text and Table S9 are from analyses using the normal prior distributions of both the Colobinae / Cercopithecinae TMRCA and the *Macaca* / Afro-papionin TMRCA. We viewed this as the most conservative approach, as they resulted in date estimates with the widest 95% confidence intervals.

Papio / Theropithecus

Fossils with the distinctive dental morphology characteristic of *Theropithecus* are first found at ~3.9 Ma [S13], and it is likely that the two lineages diverged some time just prior to that first appearance. Therefore, this node was given a lognormal distribution with an offset of 3.8 Ma and a log (standard deviation) of 0.5 (median divergence = 4.8 Ma, 95% intervals = 6.1–4.2 Ma). This range also corresponds to estimates of the divergence of these two taxa inferred from other molecular datasets [S5, S9]. Lognormal distributions are recommended for the calibration of nodes where a minimum bound is well-known but the upper bound is less certain, and because they assign the highest point probability of the age of a node to be only a little older than the earliest fossil representative of a lineage [S14].

References Cited:

S1. Goodman M, Barnabus J, Matsuda G, Moore GW (1971) Molecular evolution in the descent of man. Nature 233: 604–613.

S2. Delson E, Tattersall I, van Couvering JA, Brooks AS (2000) Encyclopedia of Human Evolution and Prehistory. New York, NY: Garland. 800 p.

S3. Jablonski NG (2002) Fossil Old World monkeys: The Late Neogene radiation. In: Hartwig WC, editor. The Primate Fossil Record. Cambridge: Cambridge University Press. pp. 255–299.

S4. Benefit BR, McCrossin ML (2002) The Victoriapithecidae, Cercopithecoidea. In: Hartwig WC, editor. The Primate Fossil Record. Cambridge: Cambridge University Press. pp. 241–253.

S5. Page SL, Chiu C, Goodman M (1999) Molecular phylogeny of Old World monkeys (Cercopithecidae) as inferred from γ-globin DNA sequences. Mol Phylogenet Evo 13: 348–359.

S6. Raaum RL, Sterner KN, Noviello CM, Stewart CB, Disotell TR (2005) Catarrhine primate divergence dates estimated from complete mitochondrial genomes: concordance with fossil and nuclear DNA evidence. J Hum Evol 48: 237–257.

S7. Sterner KN, Raaum RL, Zhang YP, Stewart CB, Disotell TR (2006) Mitochondrial data support an odd-nosed colobine clade. Mol Phylogenet Evol 40: 1–7.

S8. Ting N (2008) Mitochondrial relationships and divergence dates of the African colobines: evidence of Miocene origins for the living colobus monkeys. J Hum Evol 55: 312–325.

S9. Hodgson JA, Sterner KN, Matthews LJ, Burrell AS, Jani RA et al. (2009) Successive radiations, not stasis, in the South American primate fauna. Proc Natl Acad Sci U.S.A. 106: 5534–5539.

S10. Perelman P, Johnson WE, Roos C, Seuánez HN, Horvath JE, et al. (2011) A molecular phylogeny of living primates. PLoS Genet 7(3): e1001342. doi:10.1371/journal.pgen.1001342. Accessed 20 June 2012.

S11. Roos C, Zinner D, Kubatko LS, Schwarz C, Yang M, et al. (2011) Nuclear versus mitochondrial DNA: Evidence for hybridization in colobine monkeys. BMC Evolutionary Biology 11:77. Accessed 20 June 2012.

S12. Steiper ME, Young NM (2006) Primate molecular divergence dates. Mol Phylogenet Evol 41: 384–394.

S13. Frost SR (2007) African Pliocene and Pleistocene cercopithecid evolution and global climactic change. In: Bobe R, Alemseged Z, Behrensmeyer AK, editors. Hominin Environments in the East African Pliocene: an Assessment of the Faunal Evidence. Dordrecht: Springer. pp. 51–76.

S14. Ho SYW, Phillips MJ (2009) Accounting for calibration uncertainty in phylogenetic estimation of evolutionary divergence times. Syst Biol 58: 367–380.
